# Guide Extension Catheter-Facilitated Reverse Controlled Antegrade and Retrograde Tracking for Retrograde Recanalization of Chronic Total Occlusion

**DOI:** 10.1155/2021/6690452

**Published:** 2021-01-19

**Authors:** Rohit Mody, Debabrata Dash, Bhavya Mody, Aditya Saholi

**Affiliations:** ^1^Department of Cardiology, MAX Super Specialty Hospital, Bathinda, Punjab, India; ^2^Department of Cardiology, Zulekha Hospital, AL Zahra Street, Sharjah 457, UAE; ^3^Department of Medicine, Kasturba Medical College, Manipal, Karnataka, India; ^4^Department of Medicine, Irwin Hospital, Surja Ram Market, Malout, Punjab, India

## Abstract

**Background:**

In recent years, the retrograde approach has become a common practice in the treatment of chronic total occlusion (CTO) of coronary ostium which is arising abnormally and has an ambiguous proximal cap. In this case report, we report a case of retrograde percutaneous coronary intervention (PCI) done successfully on an abnormally originating artery which was guideliner assisted. *Case Presentation*. A 65-year-old gentleman with a history of hypertension, diabetes, and PCI presented to us with angina. Physical examination, electrocardiography (ECG), and echocardiography were done. Coronary angiography (CAG) revealed a normal left anterior descending artery (LAD), an anomalous circumflex (CX) artery arising from the right cusp. The abnormal CX had an implanted stent from which the abnormal right coronary artery (RCA) was arising and had a CTO. It also revealed the retrograde filling of distal RCA through grade 2 Werner collateral channels (CCs) from the LAD, a long CTO segment with a distal cap at the bifurcation. PCI of an RCA-CTO was scheduled utilizing a primary retrograde strategy, since antegrade ostium was abnormal in origin, and the patient was previously stented across the origin. The retrograde wire was externalized, and the procedure was completed with 3 overlapping drug-eluting stents (DESs). We used a guideliner which also assisted in the capture of retrograde corsair during the retrograde procedure of CTO [assisted reverse controlled antegrade and retrograde tracking (CART)]. These measures helped us to complete the CTO intervention successfully.

**Conclusion:**

The antegrade crossing is the most common approach to CTOs. However, it is sometimes difficult to penetrate the proximal hard ambiguous cap with guidewires, especially in the case of CTOs of anomalous coronary arteries because of a lack of support. Herein, we describe an iteration of reverse CART technique using a guide extensor catheter to facilitate externalizing the retrograde wire from false to true lumen.

## 1. Introduction

PCI of CTO has a lower success rate than PCI of nonoccluded coronary artery [1, 2]. In 1990, the first retrograde approach technique was applied for CTO via a degenerated saphenous vein graft. Over the next 2 decades, septal collateral branches were considered to be novel access for the retrograde approach [3]. With the emergence of new equipment and important iterations, this approach has become safer, faster, and more successful [4]. In this case report, we report a case of retrograde PCI done successfully on an abnormally originating artery which was guideliner assisted. Mozid et al. similarly described the utility of a guideliner catheter in retrograde PCI of a CTO with reverse CART—the “Capture” technique [5].

## 2. Case Presentation

### 2.1. History

A 65-year-old gentleman presented with Canadian Cardiovascular Society (CCS) Class III angina despite being on optimal medical therapy, including beta-blocker (Metocard XL 100 mg OD), long-acting nitrates (Nitrocontin 6.4 mg BD), calcium channel blocker (Dilzem SR 120 mg OD), and Ranolazine (Rancad 1 g OD). In addition to that, the patient used to take sublingual nitrates as and when required for angina. He was a known case of hypertension, diabetes, and a post-PCI of the proximal segment of an anomalous CX artery arising from the right cusp. The implanted stent was covering the abnormal origin of RCA from CX which had a CTO. He was also on dual antiplatelet therapy (DAPT) with aspirin 75 mg OD and Clopidogrel 75 mg OD as a post-PCI regimen. Also, the patient was on an oral hypoglycemic agent (Oxramet 5 mg/1000 mg OD) with poorly controlled diabetes (HbA1c: 8%).

### 2.2. Differential Diagnoses

In this patient, acute coronary syndrome was most suspected, followed by myocarditis, pericarditis, and stress cardiomyopathy.

### 2.3. Investigations

**Figure 1:**
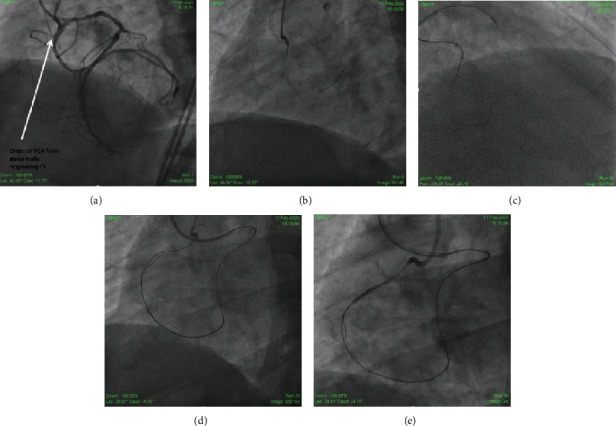
(a) Retrogradely filling abnormal RCA originating from abnormally originating CX. (b) POT done in CX stent. (c) Sion blue and Corsair crossed retrogradely. (d) Knuckle pilot wire reaching proximal RCA subintimaly. (e) Guideliner assisted creation of common space antegradely.

The patient's routine blood investigations were sent. An ECG revealed normal sinus rhythm at a rate of 62 beats/min without abnormal QS wave patterns. An echocardiogram revealed normal overall left ventricular function. Troponin I and CPK-MB were normal. The diagnostic CAG revealed a normal LAD and anomalous CX arising from the right coronary cusp along with a CTO in the proximal segment of the anomalous RCA. This RCA was arising from the proximal segment of abnormally originating CX artery. It also revealed the retrograde filling of distal RCA through grade 2 Werner CCs from the LAD, a long CTO segment with a distal cap at the bifurcation (Figure 1(a)). Therefore, the PCI of an RCA-CTO was scheduled utilizing a primary retrograde strategy.

### 2.4. Management

**Figure 2:**
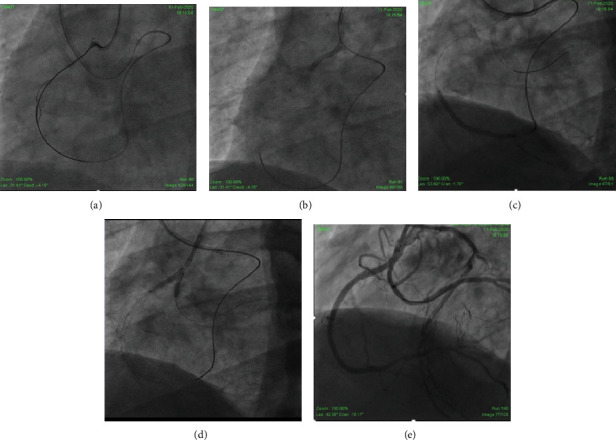
(a) RG3 wire externalized with the help of guideliner. (b) Corsair taken back. (c) 3 stents deployed sequentially. (d).Final proximal kissing. (e) Final result.

**Figure 3:**
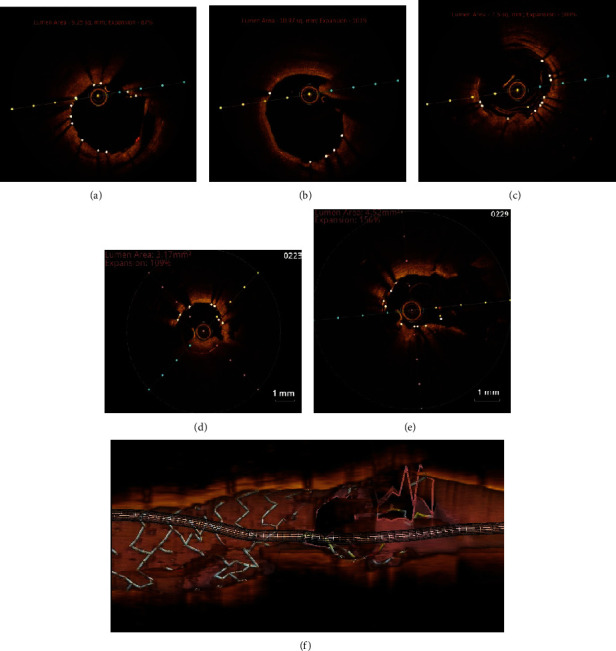
(a) RCA showing intimal tear within stented segment. (b) Proximal edge with no edge dissection. (c) RCA—distal edge. (d) CX—tissue prolapse. (e) CX bifurcation view—no pinching. (f). CX bifurcation view—longitudinal.

Vascular access was obtained using bilateral femoral 7-Fr long sheaths. Particularly, 7-Fr Hyperion AL 1 (Asahi Intecc, Aichi, Japan) and 7-Fr extra backup (EBU) 3.5 (Medtronic, USA) with side holes were utilized to engage the CX arising from the right cusp and the LAD, respectively. A workhorse wire was advanced into the distal abnormal CX. A 3.5 × 15 mm noncompliant (NC) balloon (Mozec™ NC PTCA Balloon) was used to postdilate the previously implanted stent for smooth navigation of gears (Figure 1(b)). A Sion wire (Asahi Intecc, Aichi, Japan) and 150 cm Corsair® microcatheter (Asahi Intecc, Aichi, Japan) were advanced through the septal CC into the posterior descending artery progressing easily until the distal cap of the occlusion (Figure 1(c)). Similarly, the escalation of the retrograde wire was done. The initial Sion blue was exchanged with Pilot 200 (Abbott Vascular International, Diegem, Belgium), and an attempt to navigate it through the distal cap was unsuccessful. Hence, the distal cap was crossed with a knuckle which could be advanced in subintimal space from distal to proximal RCA allowing delivery of the Corsair® (Figure 1(d)). Unable to progress further, a decision was made to introduce an anterograde subintimal track through the struts of an already deployed CX stent, from which abnormal RCA was arising, to meet the retrograde wire. A Gaia 2 (Asahi Intecc, Aichi, Japan) was taken through a Finecross microcatheter (Terumo Corp., Tokyo, Japan), escalated to Gaia 3, and finally Conquest Pro 12 (Asahi Intecc, Aichi, Japan). This entered the subintimal space anterogradely but was unable to meet the retrograde wire. To optimize the subintimal space, it was dilated with a 3 × 15 mm NC balloon. When this was also futile, a guide extension catheter (Guideliner [Vascular Solutions, Minneapolis, MN, USA]) was positioned in the antegrade subintimal space which created a target lumen enabling rapid reconnection with the true lumen from the retrograde wire (Figure 1(e)). Once the wire engaged the antegrade guide, the procedure became standard reverse CART with RG3 wire (Asahi Intecc, Aichi, Japan) externalized (Figure 2(a)). Following predilation with serial ballooning, 3 overlapping DESs were implanted (Figures 2(b) and 2(c)). The final proximal RCA stent, a 3.5 × 18 mm DES (cobalt-chromium everolimus-eluting stent; Xpedition; Abbott Vascular, Santa Clara, California), was deployed with T and protrusion (TAP) technique touching the CX stent. Final kissing was done with 3 × 15 mm NC balloon (Mozec™ NC PTCA Balloon) in the CX and withdrawn 3.5 × 18 mm stent balloon at 12 atm after the sequential dilatation of respective balloons to 18 atm (Figure 2(d)). The final angiographic result was excellent (Figure 2(e)). Finally, an optical coherence tomography (OCT) run was taken to see the final optimization which showed optimized stent deployment, no edge dissection, good bifurcation angle, and no major complication (Figure 3).

### 2.5. Follow-Up

The patient was followed up at 3-month intervals in the outpatient department. The patient is free of angina on minimal doses of beta-blocker (Metocard XL 50 mg OD) and long-acting nitrates (Imdur 30 mg OD), and there was no need for sublingual nitrates and calcium channel blockers. He had no symptoms pertaining to cardiovascular symptoms. His ECG was unremarkable, and ECHO showed EF of 60%. Blood sugar was better controlled with HbA1c of 7%.

## 3. Discussion

The most common cause of failure of PCI for CTO is the inability of wire to cross the occlusion [6, 7]. Recently, the retrograde approach through the CCs was introduced to overcome this problem, and several strategies and techniques for the retrograde approach have been developed to increase the efficacy and feasibility of this technique for CTO intervention [8–10]. Recently, Dash [4] described four iterations of reverse CART: (1) conventional reverse CART; (2) contemporary reverse CART involving the use of small antegrade balloons and more active, intentional vessel tracking and penetration; (3) assisted reverse CART involving assistance from intravascular ultrasound (IVUS), stent, guide extension catheter, etc.; and (4) shifting reverse CART. Our case describes how the optimization of the reentry space can be escalated, with the advantage of the guide extensor catheter forming a clear open target, enabling rapid advancement of the retrograde wire into the proximal true lumen. This procedure could have been assisted by IVUS and stent. Stent-facilitated reverse CART is by nature an irreversible procedure as once the stent is deployed, it cannot be removed [4, 11, 12]. Unlike a stent, the guide extension device may be removed or repositioned once there is a failure of connection between the antegrade and retrograde true lumen [4, 11]. In our case, guideliner-assisted reverse CART was done. Firstly, in our case, the origin of the artery was abnormal; hence, good antegrade preparation was not possible with only AL1 support. Hence, guideliner was used. As the lesion was already stented previously so a good preparation with semicompliant balloon dilatation had to be made to advance guideliner through struts. As we know there may be compression or collapse of subintimal space after antegrade balloon inflation making wiring the true lumen more difficult even if common subintimal space does exist. We overcame this difficulty by the use of mother and child catheter. The advantage of this technique is that we can change the position of the guideliner to capture the retrograde microcatheter which otherwise cannot be done if the stent reverse CART technique is used [11]. Also, in our case, it was decided to go retrograde as an initial strategy as there was an abnormal origin and the segment had an overlying stent making antegrade entry ambiguous.

## 4. Conclusion

To conclude, retrograde PCI using reverse CART should be the first-line option for patients of CTO and abnormally originating artery. Also, during reverse CART in these lesions, guideliner can help support antegrade preparation as well as can help in the iteration of reverse CART.

## Data Availability

The PowerPoint presentation made to support the findings of this study is included within the supplementary information file.

## Abbreviations

PCI: Percutaneous intervention
CTO: Chronic total occlusion
CX: Circumflex
CCS: Canadian Cardiovascular Society
LAD: Left anterior descending
RCA: Right coronary artery
CCs: Collateral channels
Fr: French
AL: Amplatz
NC: Noncompliant
CART: Controlled antegrade and retrograde tracking
CAG: Coronary angiography
ECG: Electrocardiogram
IVUS: Intravascular ultrasound
DAPT: Dual antiplatelet therapy
DES: Drug-eluting stent
TAP: T and protrusion
OCT: Optical coherence tomography.

## Supplementary Materials

Supplementary Materials Link to the PowerPoint presentation of the entire case, including the angiographic and OCT videos, is given in the attached supplementary file.
