# Supplementary material for: Guide Extension Catheter-Facilitated Reverse Controlled Antegrade and Retrograde Tracking for Retrograde Recanalization of Chronic Total Occlusion
Source: Case Rep Cardiol. 2021 Jan 19;2021:6690452. doi: 10.1155/2021/6690452 (PMC7840250; doi:10.1155/2021/6690452)
Supplement: Supplementary Materials — Link to the PowerPoint presentation of the entire case, including the angiographic and OCT videos, is given in the attached supplementary file. [file 6690452.f1.pptx]

## Slide 1
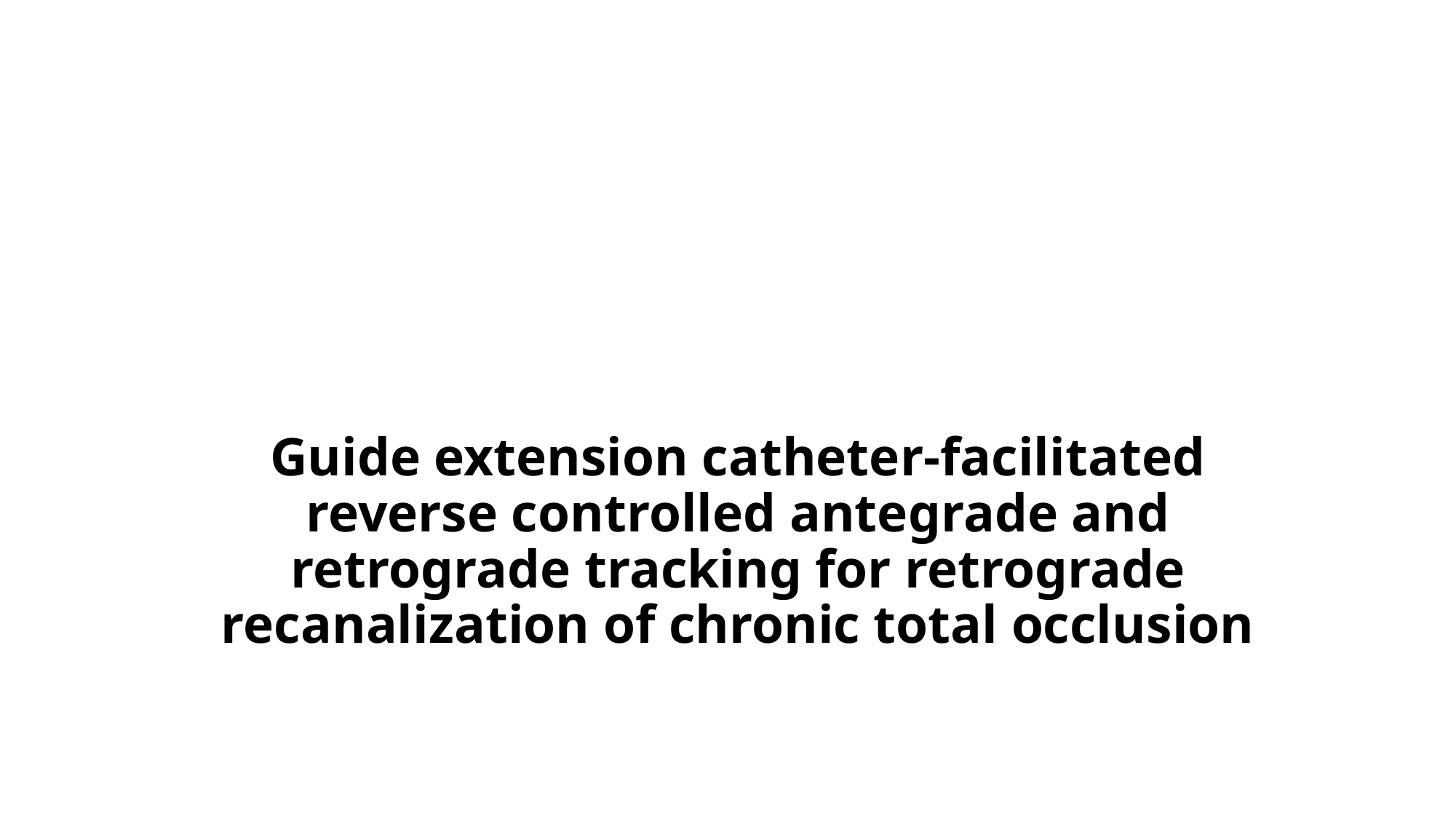

# Guide extension catheter-facilitated reverse controlled antegrade and retrograde tracking for retrograde recanalization of chronic total occlusion

## Slide 2
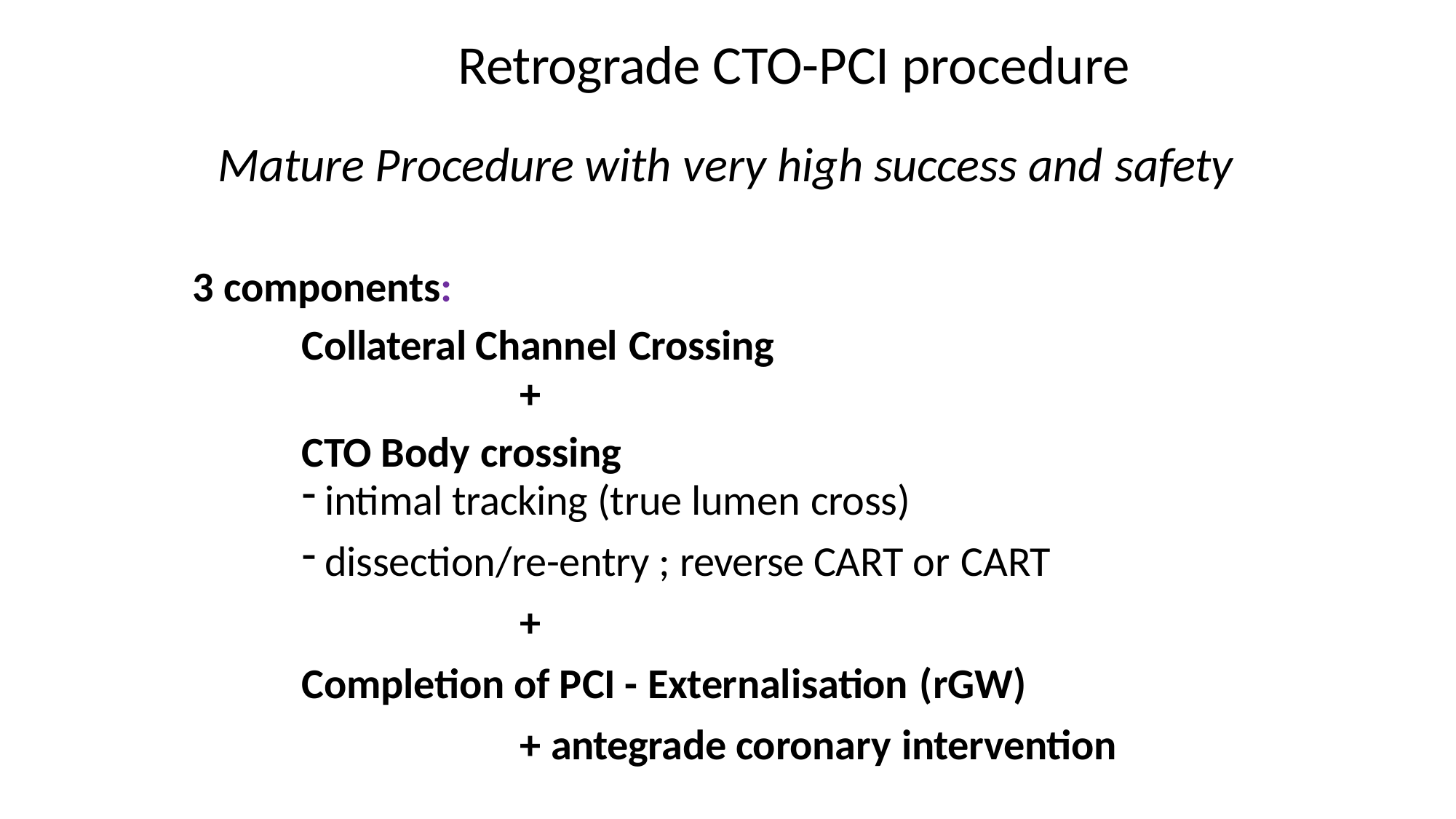

# Retrograde CTO-PCI procedure
Mature Procedure with very high success and safety
3 components:
Collateral Channel Crossing
+
CTO Body crossing
intimal tracking (true lumen cross)
dissection/re-entry ; reverse CART or CART
+
Completion of PCI - Externalisation (rGW)
+ antegrade coronary intervention

## Slide 3
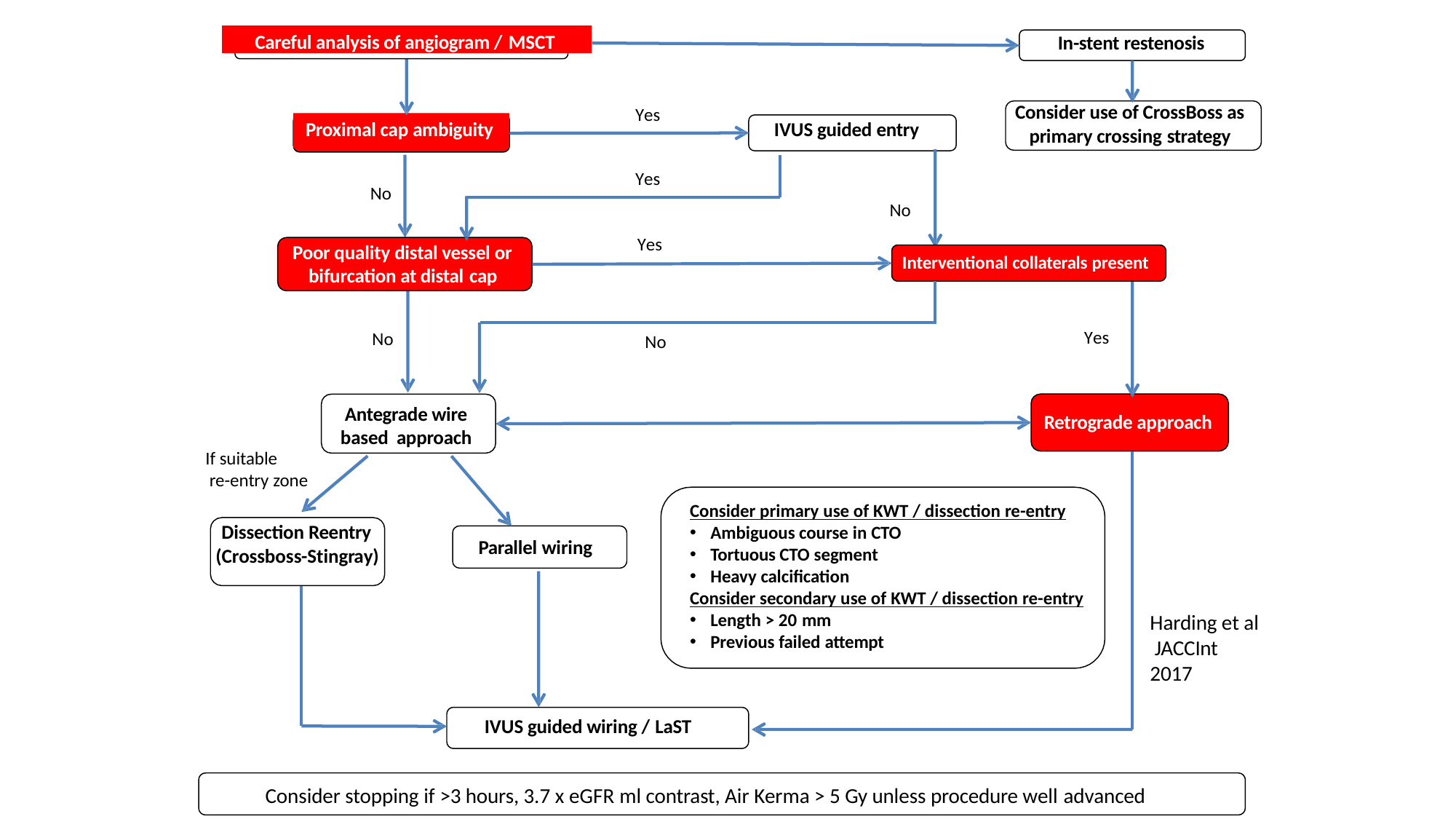

Careful analysis of angiogram / MSCT
In-stent restenosis
Consider use of CrossBoss as primary crossing strategy
Yes
Proximal cap ambiguity
IVUS guided entry
Yes
No
No
Yes
Poor quality distal vessel or bifurcation at distal cap
Interventional collaterals present
Yes
No
No
Antegrade wire based approach
Retrograde approach
If suitable
re-entry zone
Consider primary use of KWT / dissection re-entry
Dissection Reentry (Crossboss-Stingray)
Ambiguous course in CTO
Tortuous CTO segment
Heavy calcification
Parallel wiring
Consider secondary use of KWT / dissection re-entry
Length > 20 mm
Previous failed attempt
Harding et al JACCInt 2017
IVUS guided wiring / LaST
Consider stopping if >3 hours, 3.7 x eGFR ml contrast, Air Kerma > 5 Gy unless procedure well advanced

## Slide 4
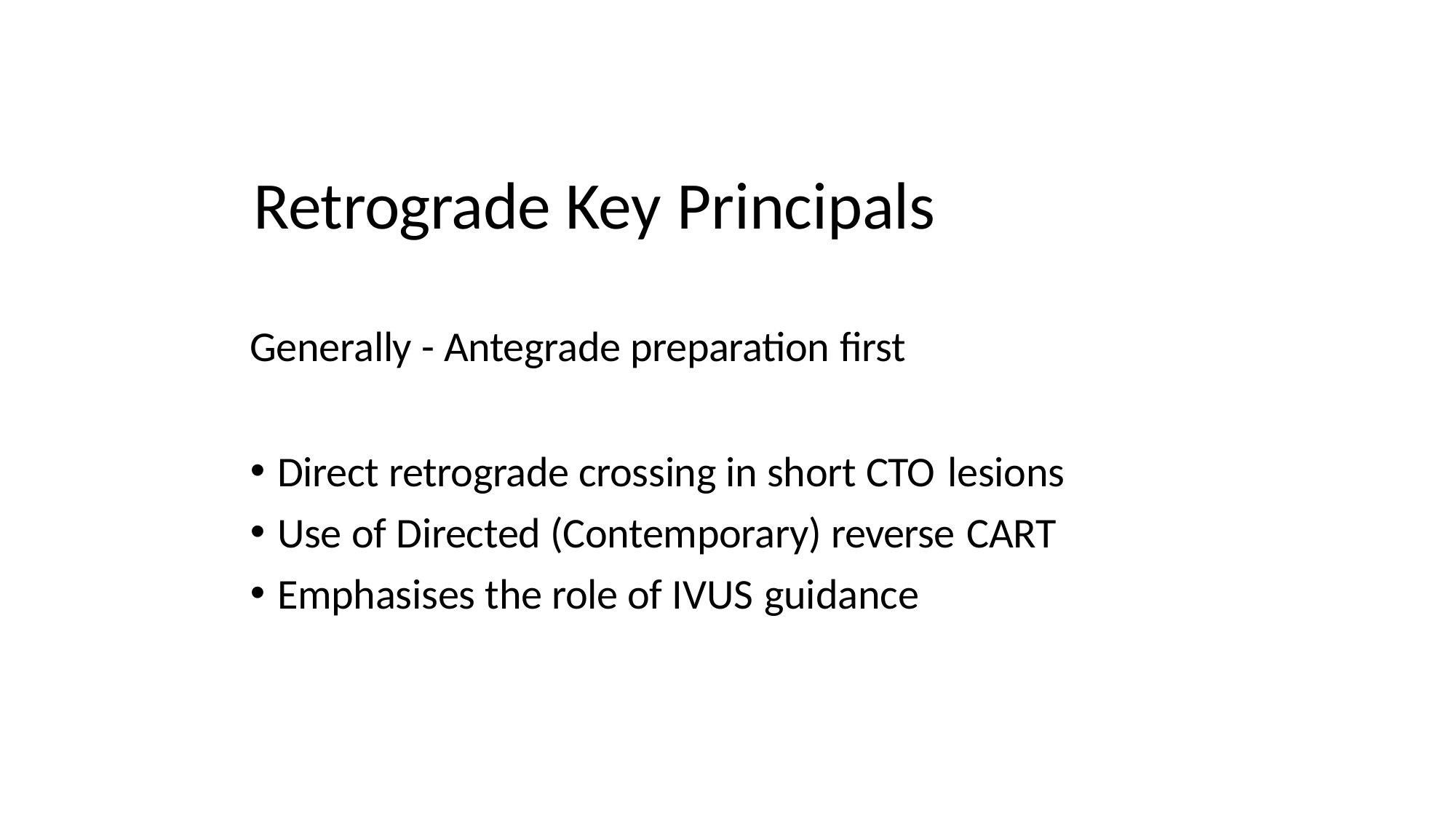

# Retrograde Key Principals
Generally - Antegrade preparation first
Direct retrograde crossing in short CTO lesions
Use of Directed (Contemporary) reverse CART
Emphasises the role of IVUS guidance

## Slide 5
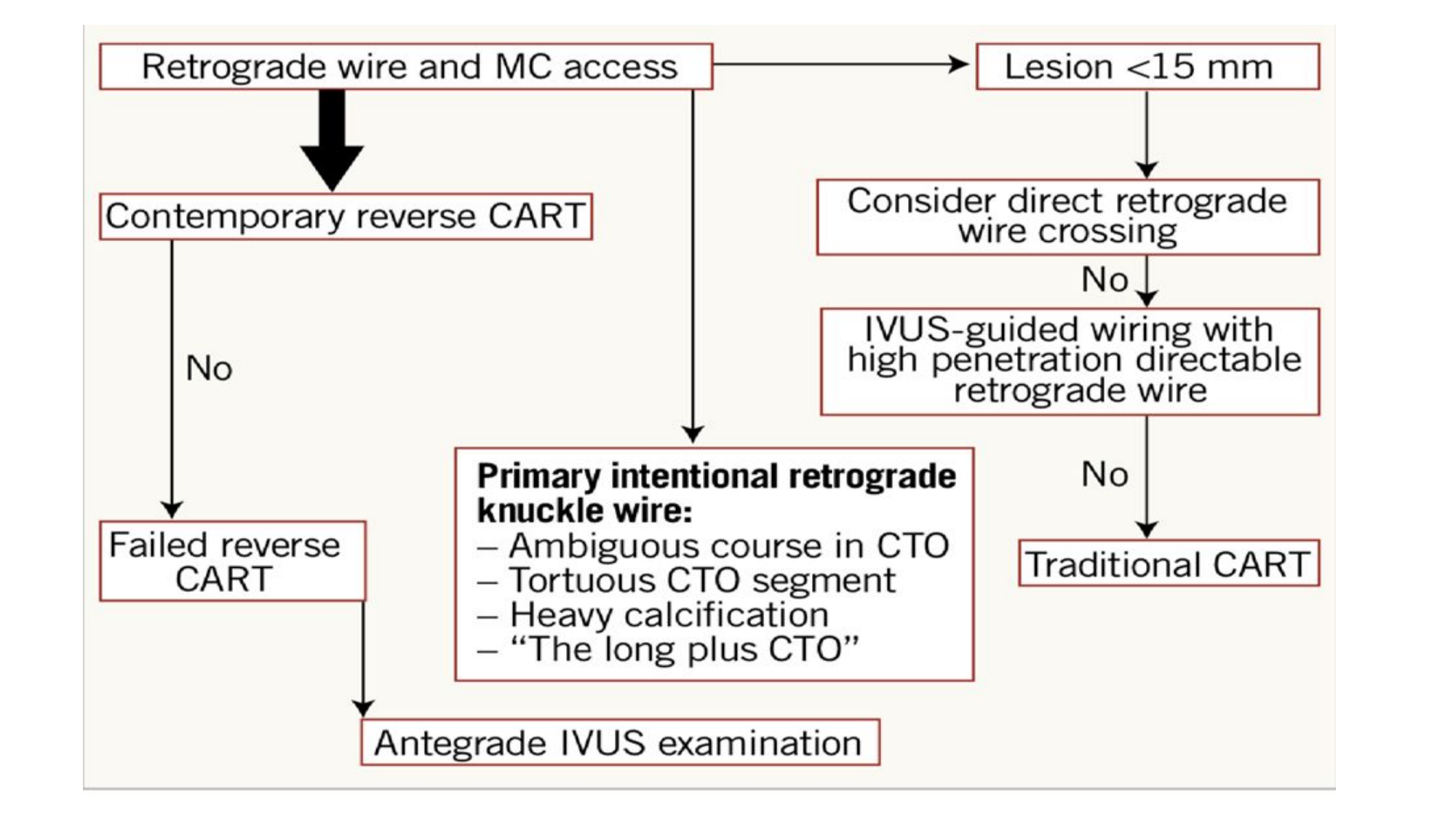

## Slide 6
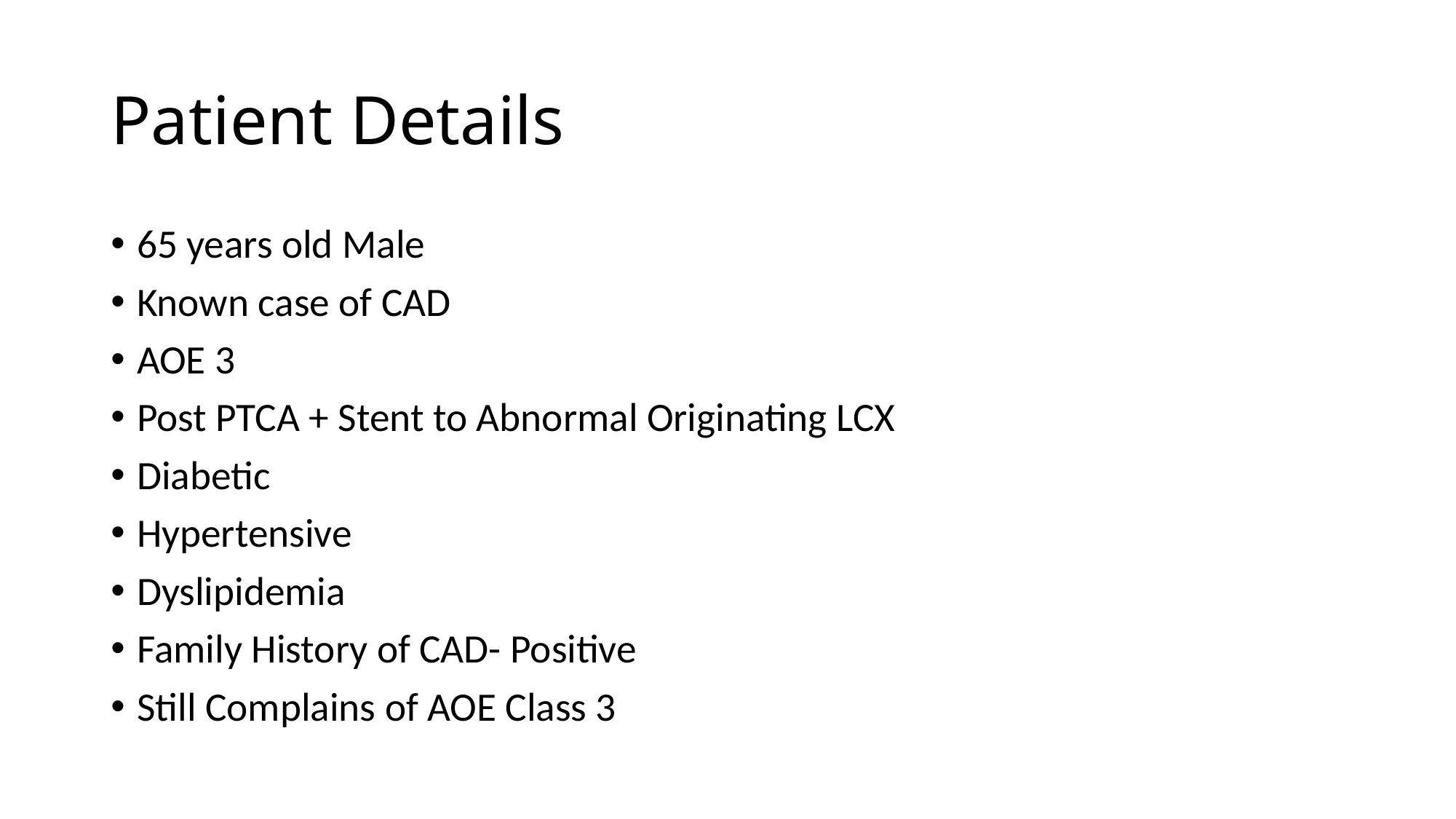

# Patient Details
65 years old Male
Known case of CAD
AOE 3
Post PTCA + Stent to Abnormal Originating LCX
Diabetic
Hypertensive
Dyslipidemia
Family History of CAD- Positive
Still Complains of AOE Class 3

## Slide 7
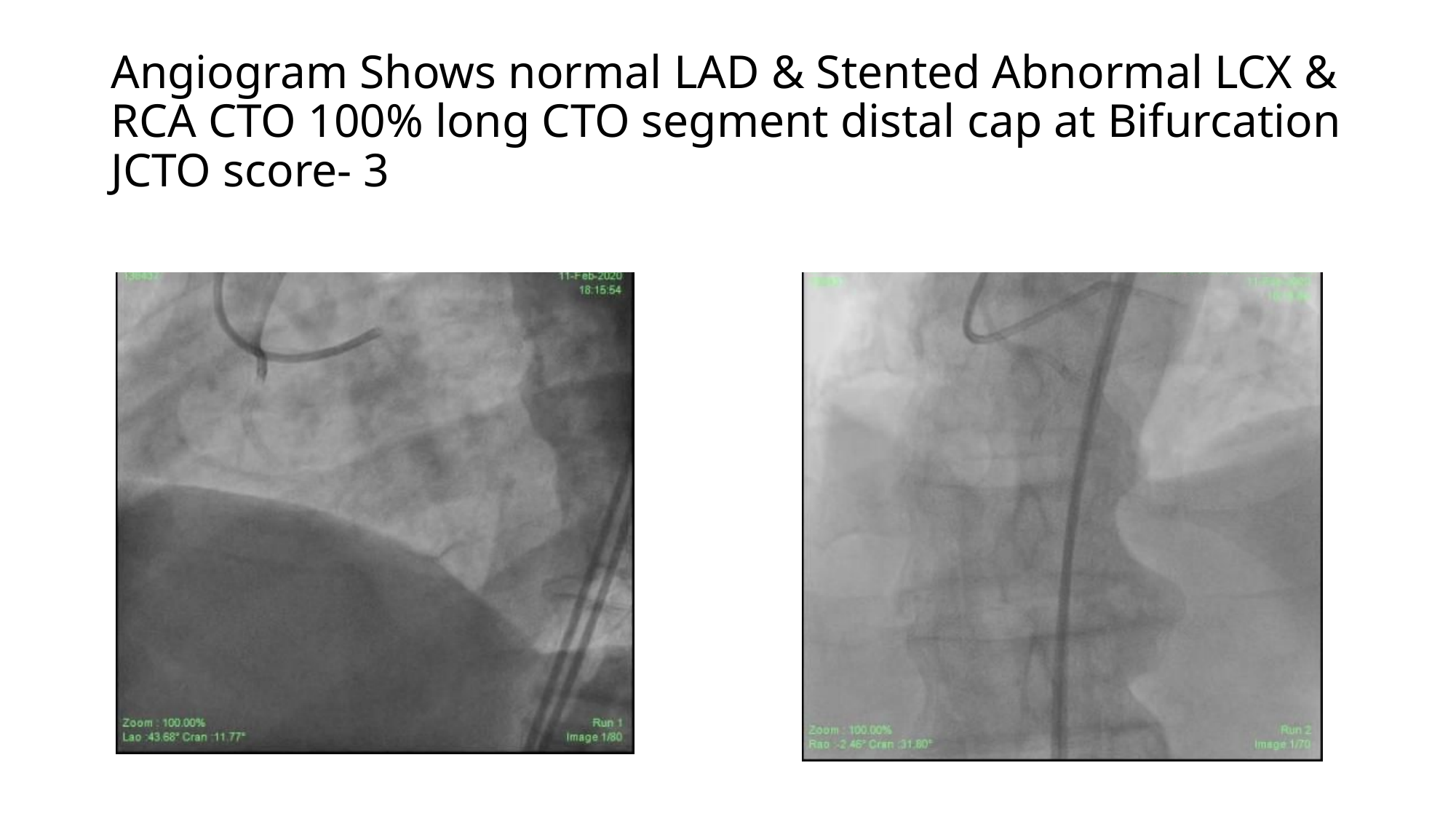

# Angiogram Shows normal LAD & Stented Abnormal LCX & RCA CTO 100% long CTO segment distal cap at Bifurcation JCTO score- 3

## Slide 8
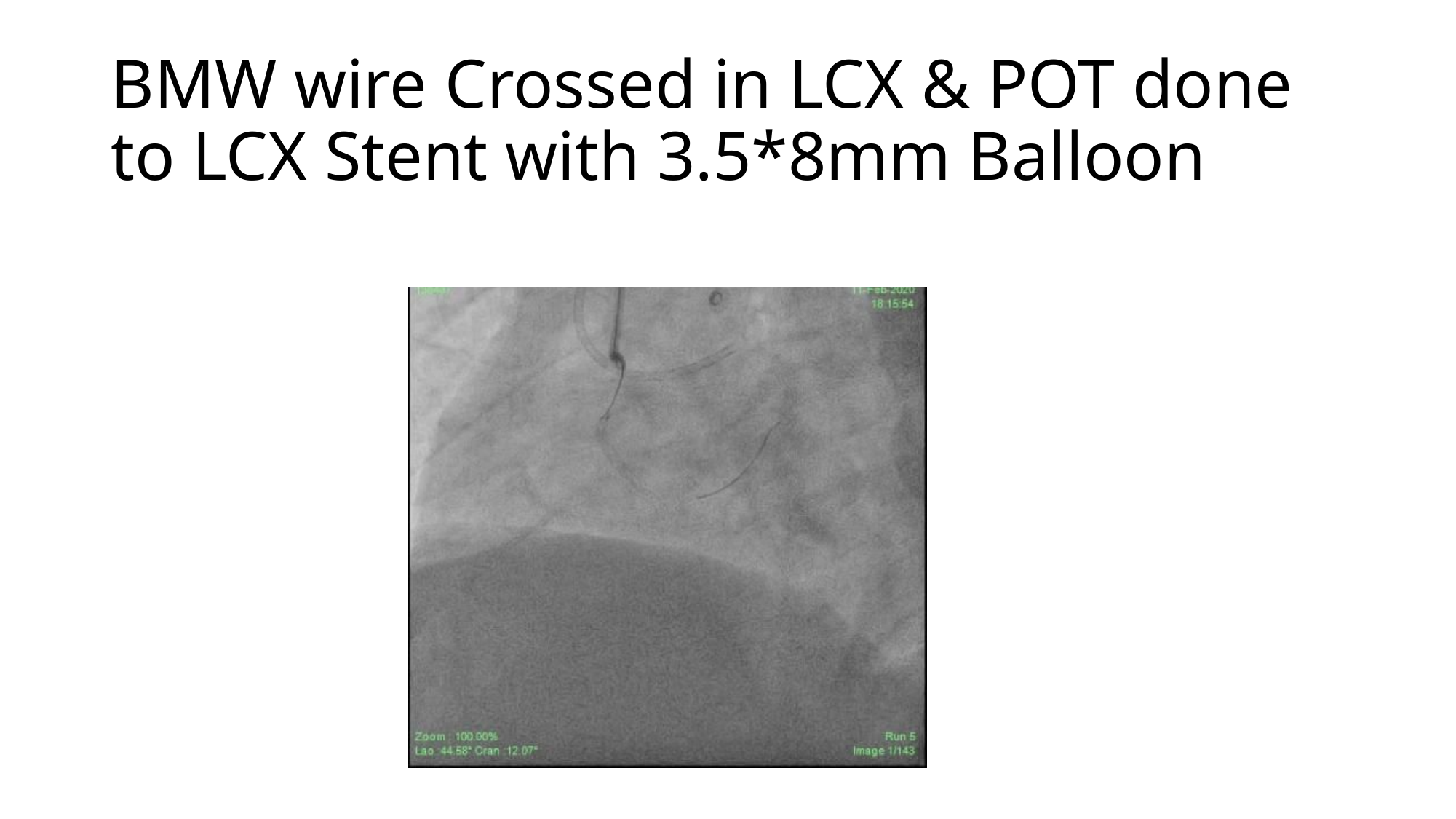

# BMW wire Crossed in LCX & POT done to LCX Stent with 3.5*8mm Balloon

## Slide 9
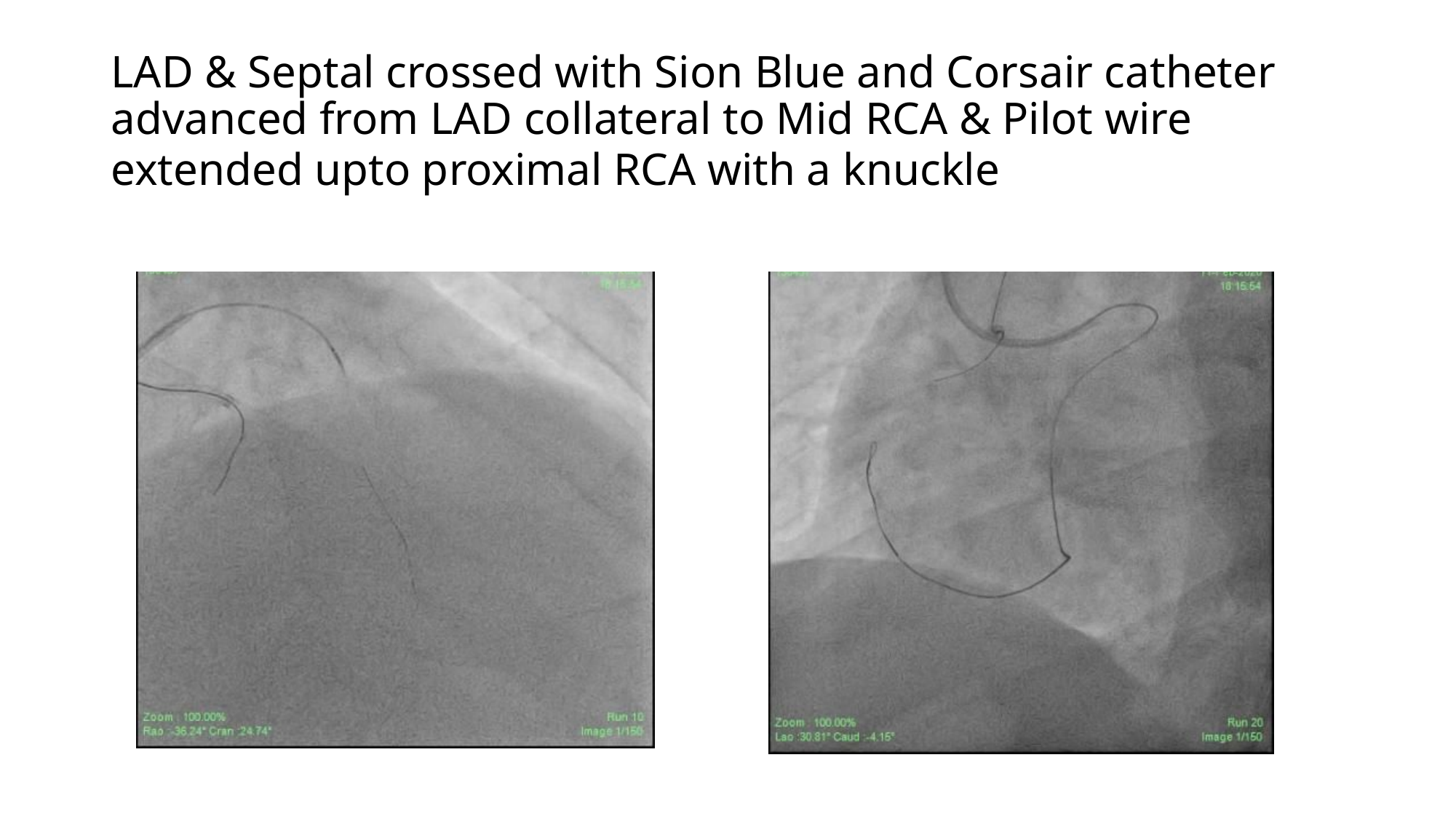

# LAD & Septal crossed with Sion Blue and Corsair catheter advanced from LAD collateral to Mid RCA & Pilot wire extended upto proximal RCA with a knuckle

## Slide 10
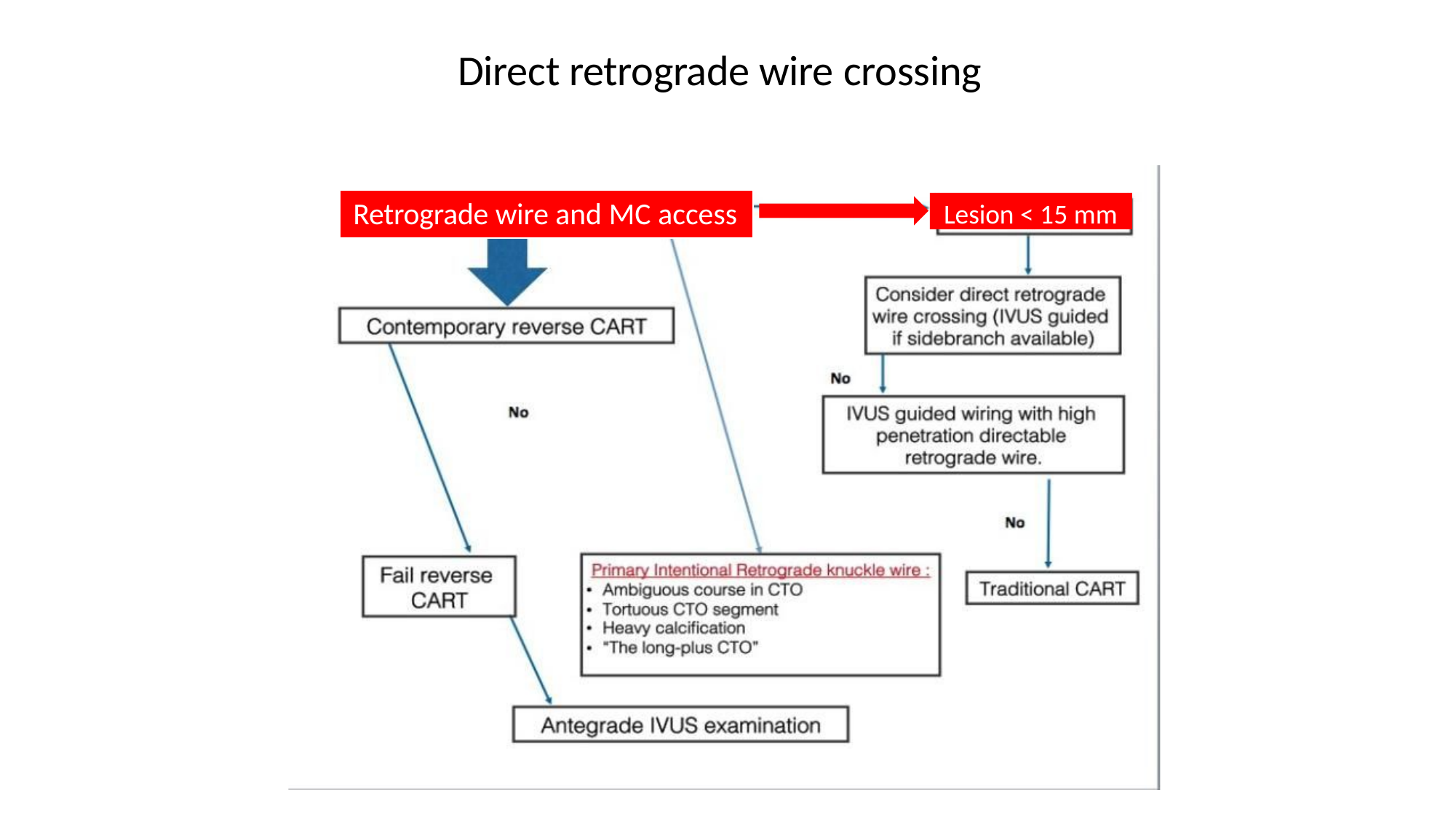

# Direct retrograde wire crossing
Retrograde wire and MC access
Lesion < 15 mm

## Slide 11
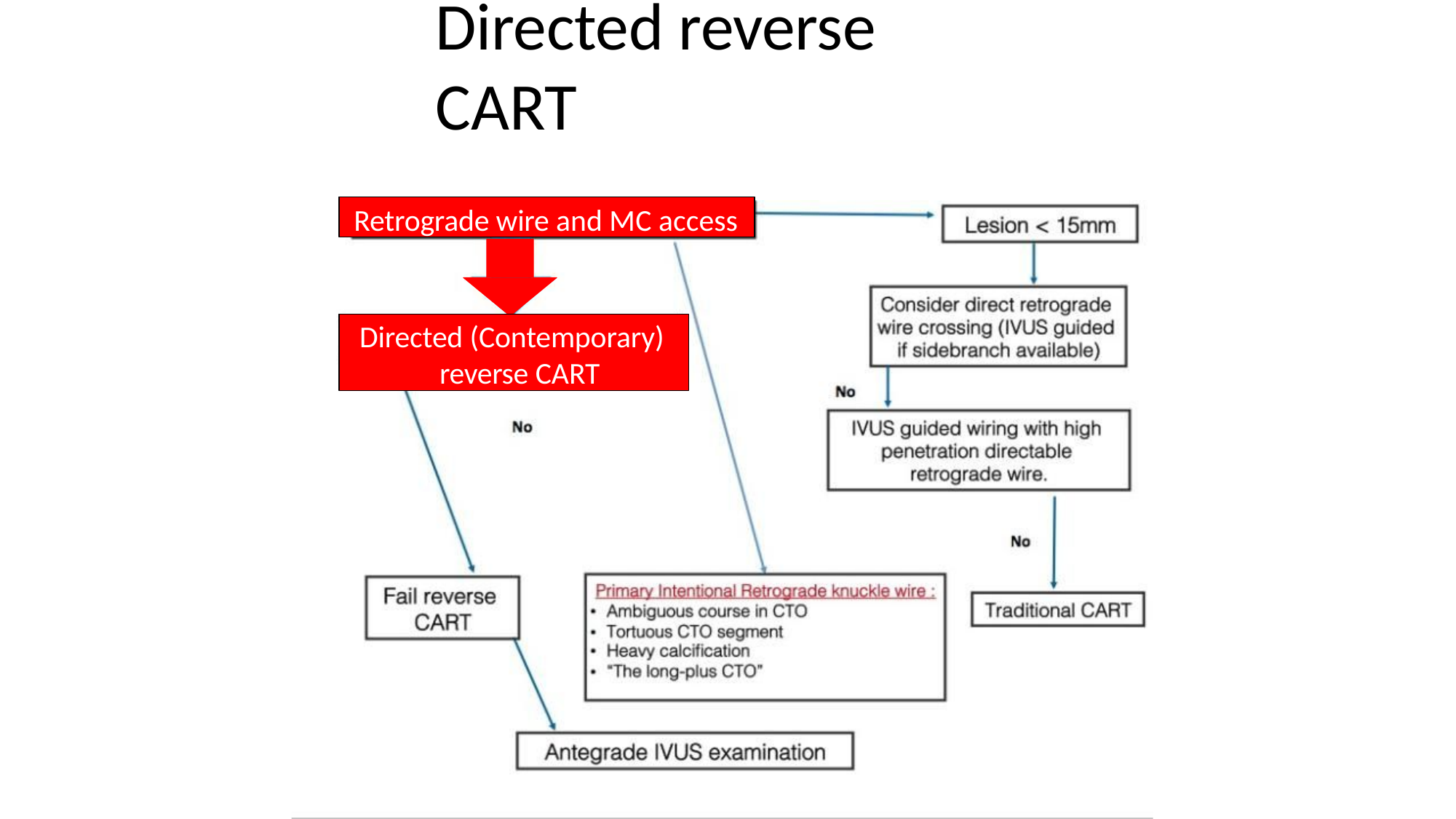

# Directed reverse CART
Retrograde wire and MC access
Directed (Contemporary) reverse CART

## Slide 12
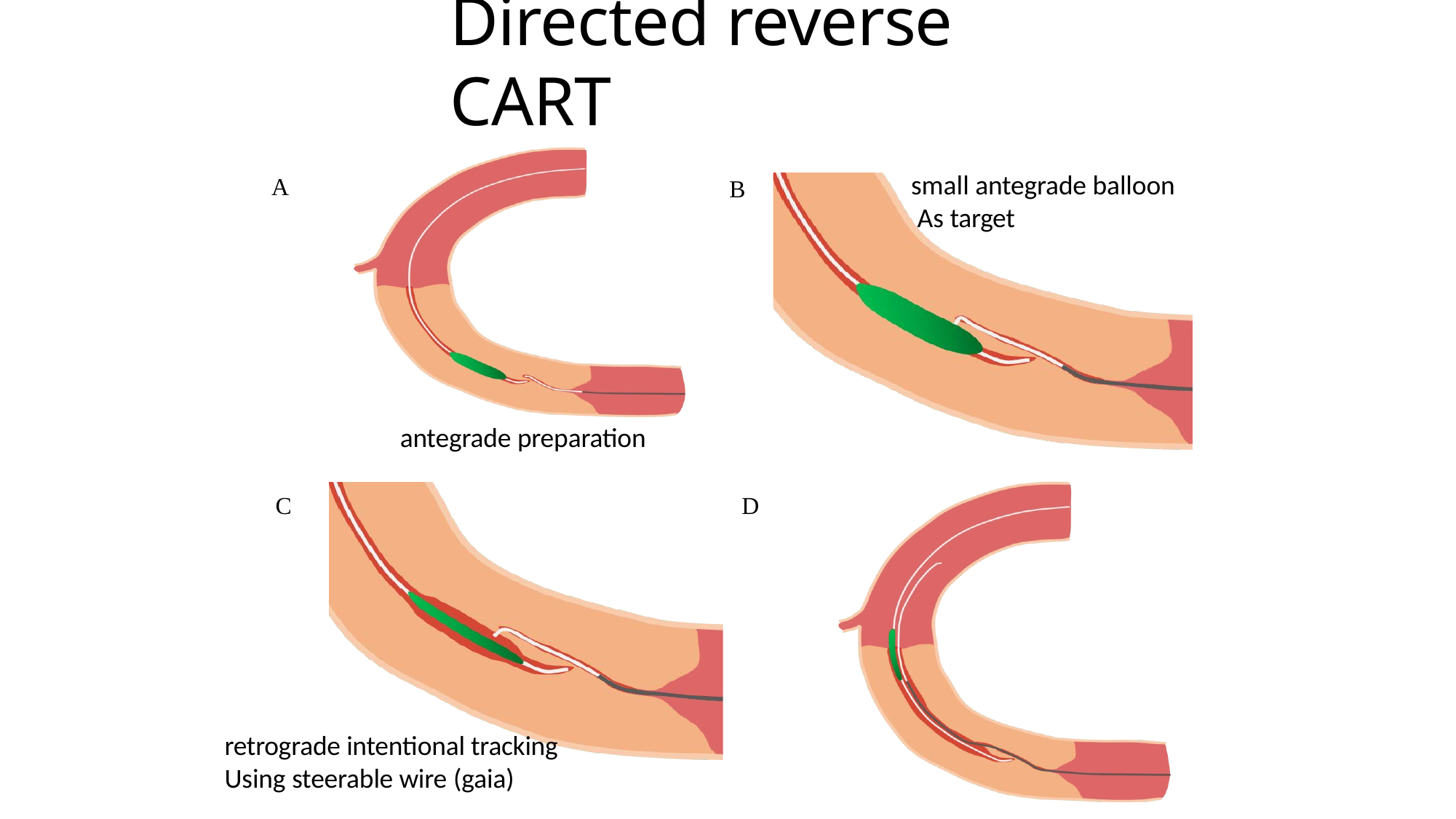

# Directed reverse CART
small antegrade balloon As target
A
B
antegrade preparation
C
D
retrograde intentional tracking Using steerable wire (gaia)

## Slide 13
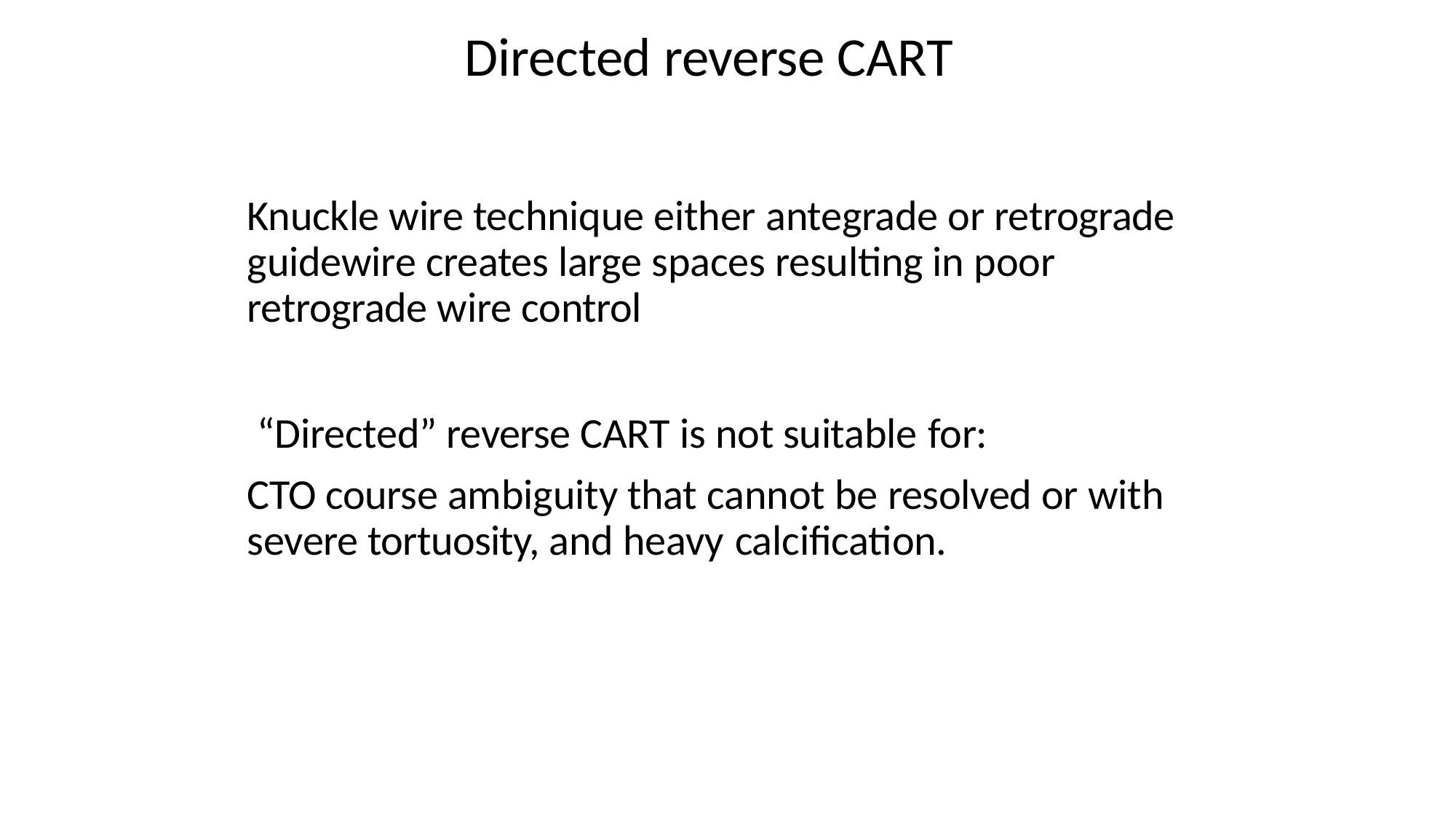

# Directed reverse CART
Knuckle wire technique either antegrade or retrograde guidewire creates large spaces resulting in poor retrograde wire control
“Directed” reverse CART is not suitable for:
CTO course ambiguity that cannot be resolved or with severe tortuosity, and heavy calcification.

## Slide 14
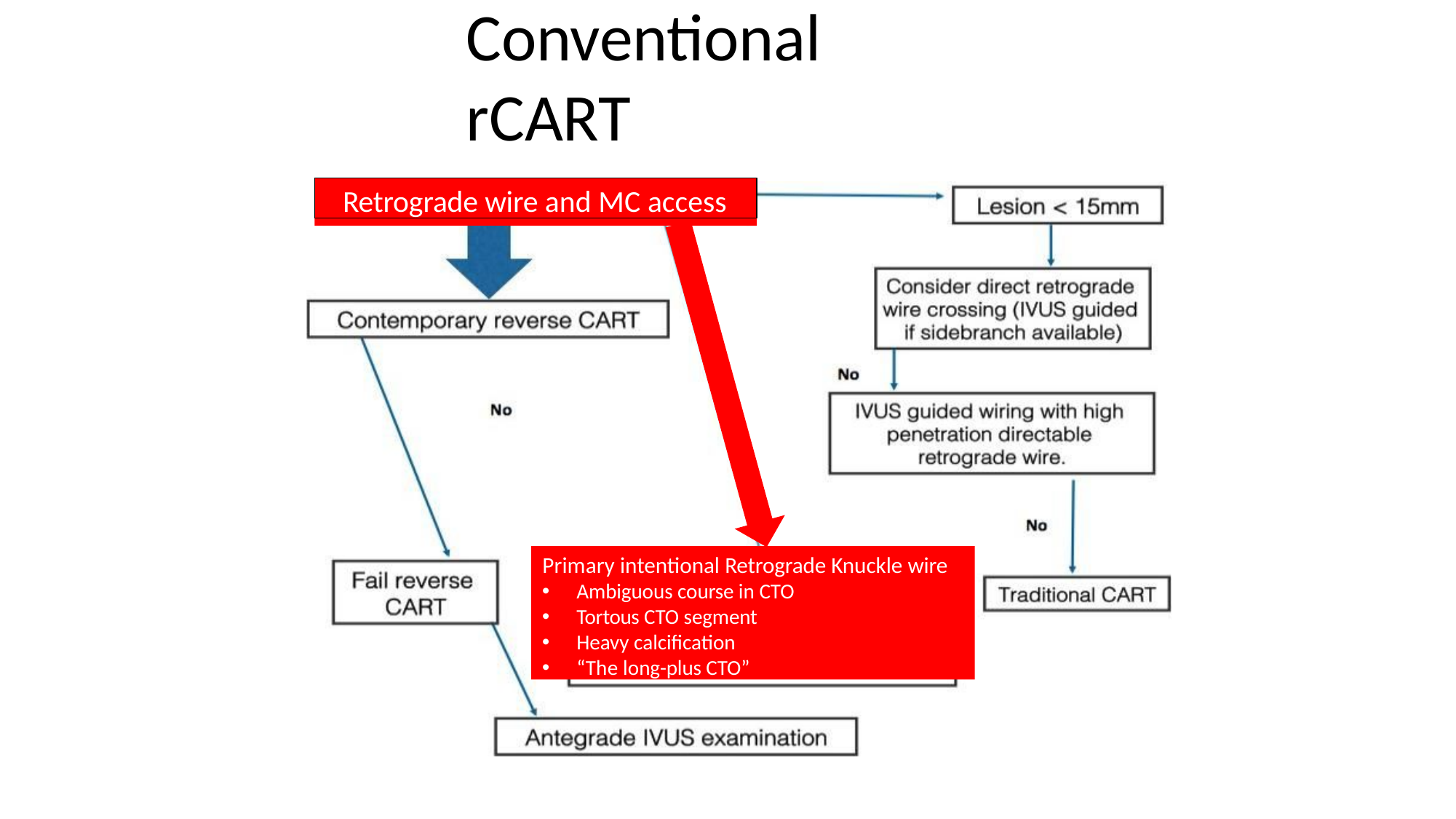

# Conventional rCART
Retrograde wire and MC access
Primary intentional Retrograde Knuckle wire
Ambiguous course in CTO
Tortous CTO segment
Heavy calcification
“The long-plus CTO”

## Slide 15
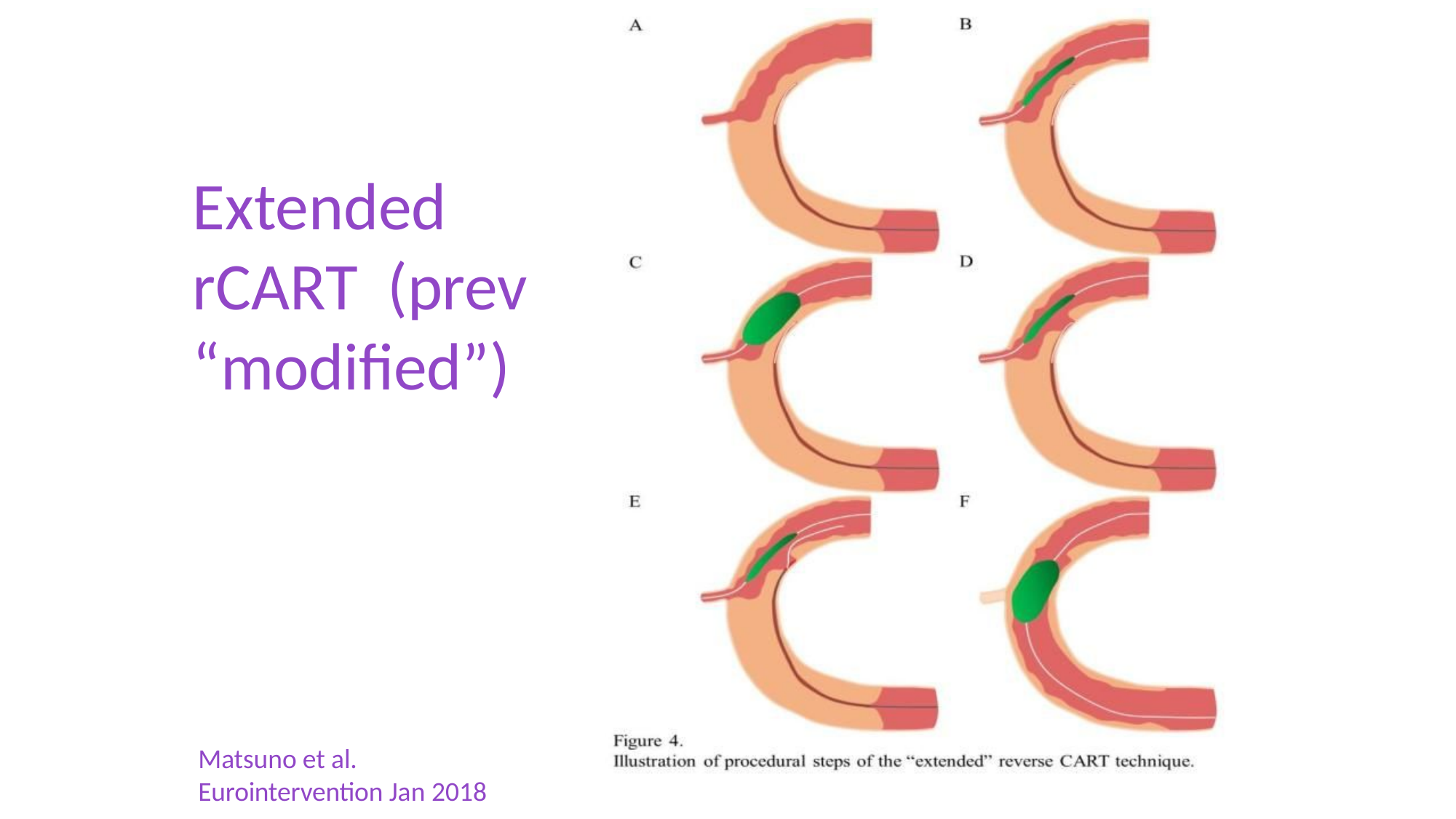

# Extended rCART (prev “modified”)
Matsuno et al. Eurointervention Jan 2018

## Slide 16
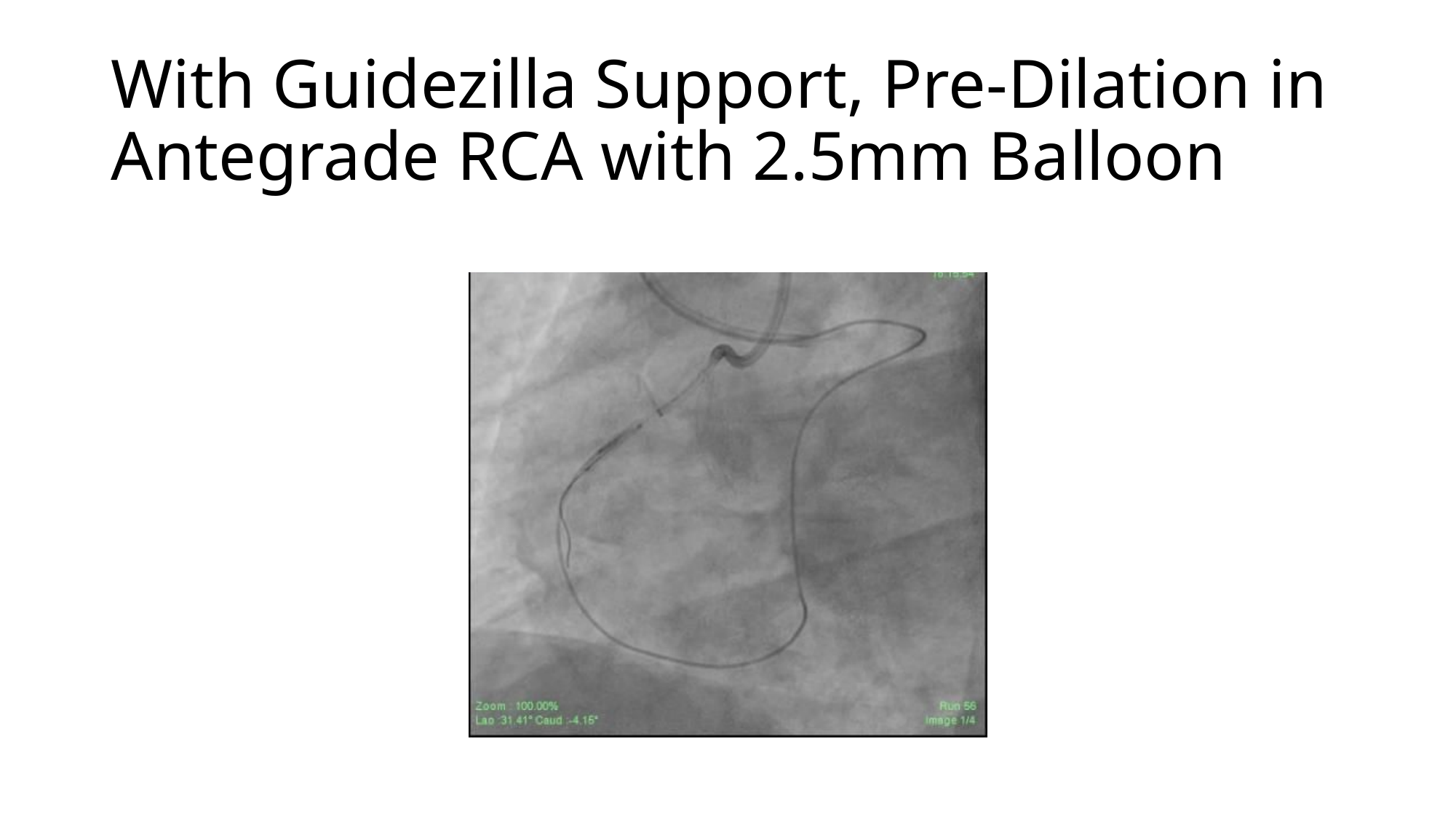

# With Guidezilla Support, Pre-Dilation in Antegrade RCA with 2.5mm Balloon

## Slide 17
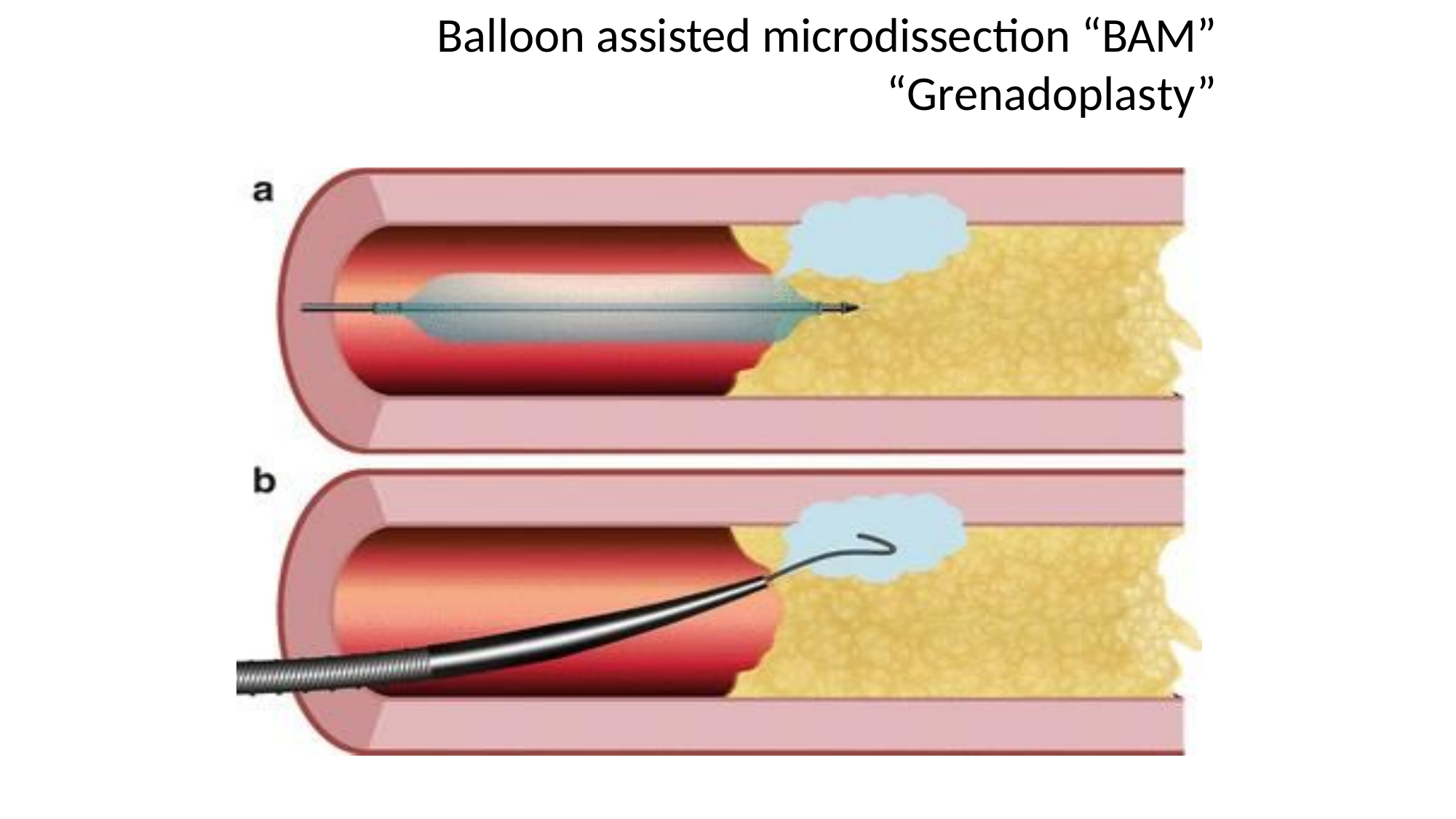

# Balloon assisted microdissection “BAM”
“Grenadoplasty”

## Slide 18
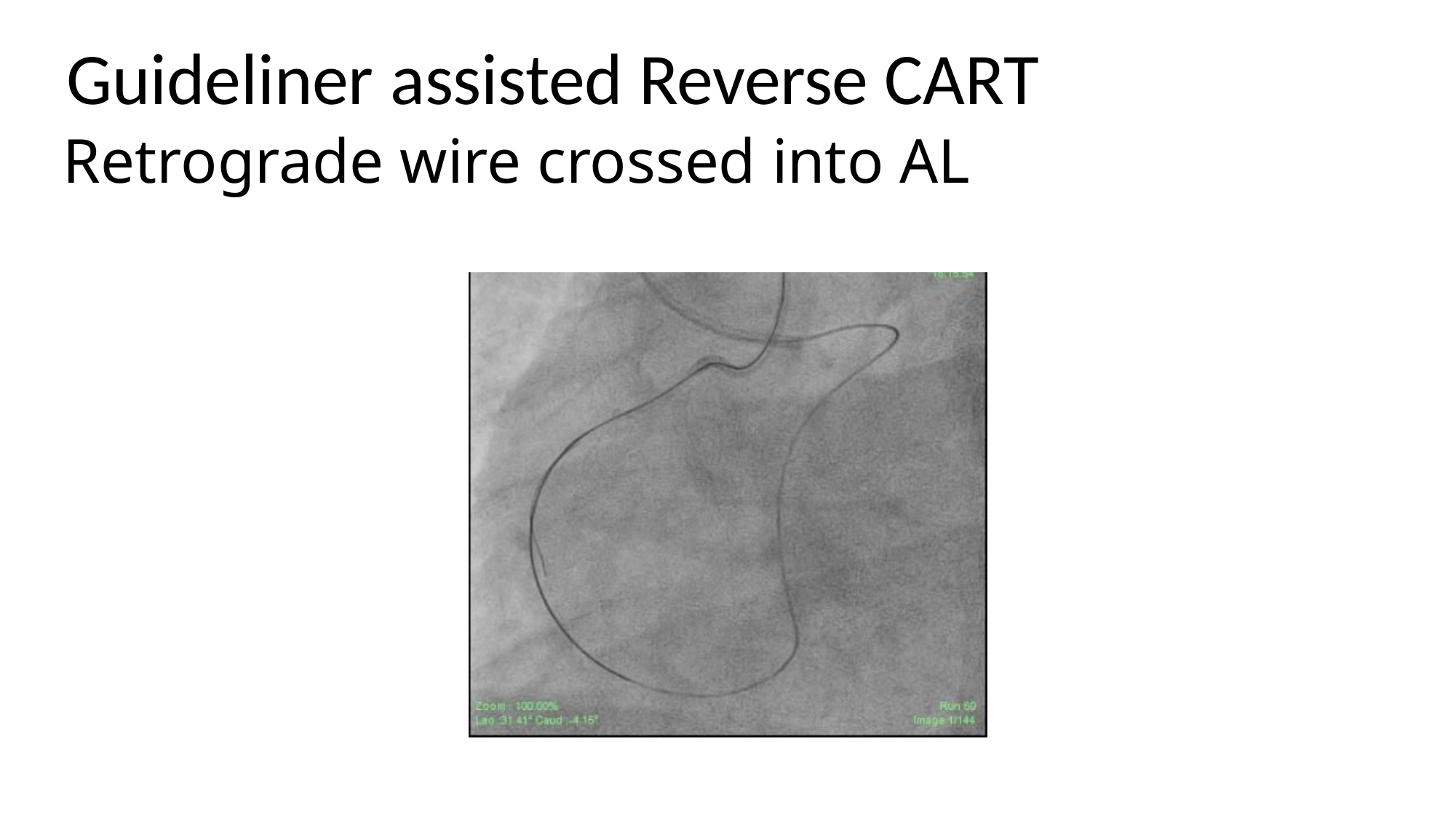

Guideliner assisted Reverse CART
# Retrograde wire crossed into AL

## Slide 19
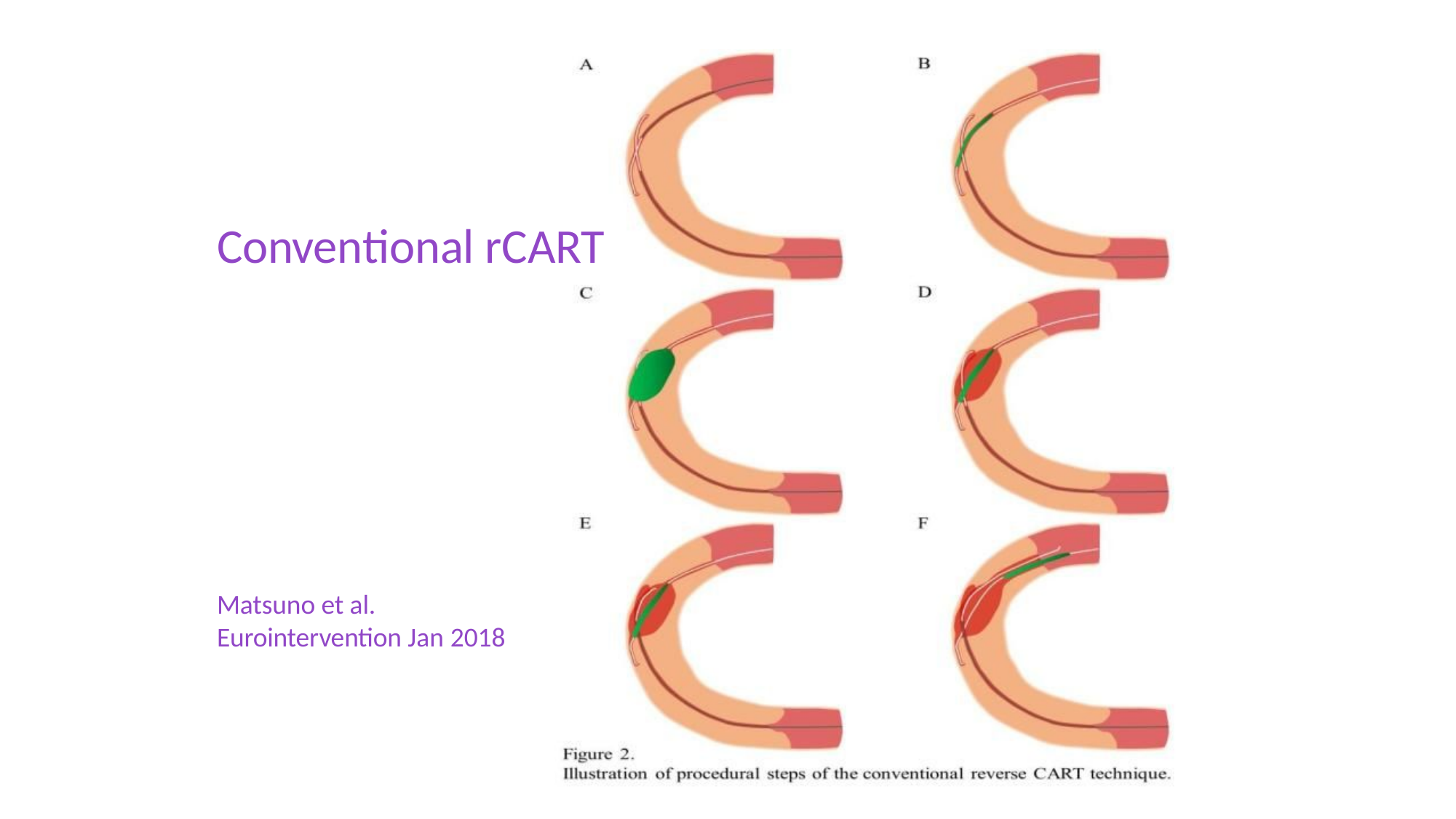

# Conventional rCART
Matsuno et al. Eurointervention Jan 2018

## Slide 20
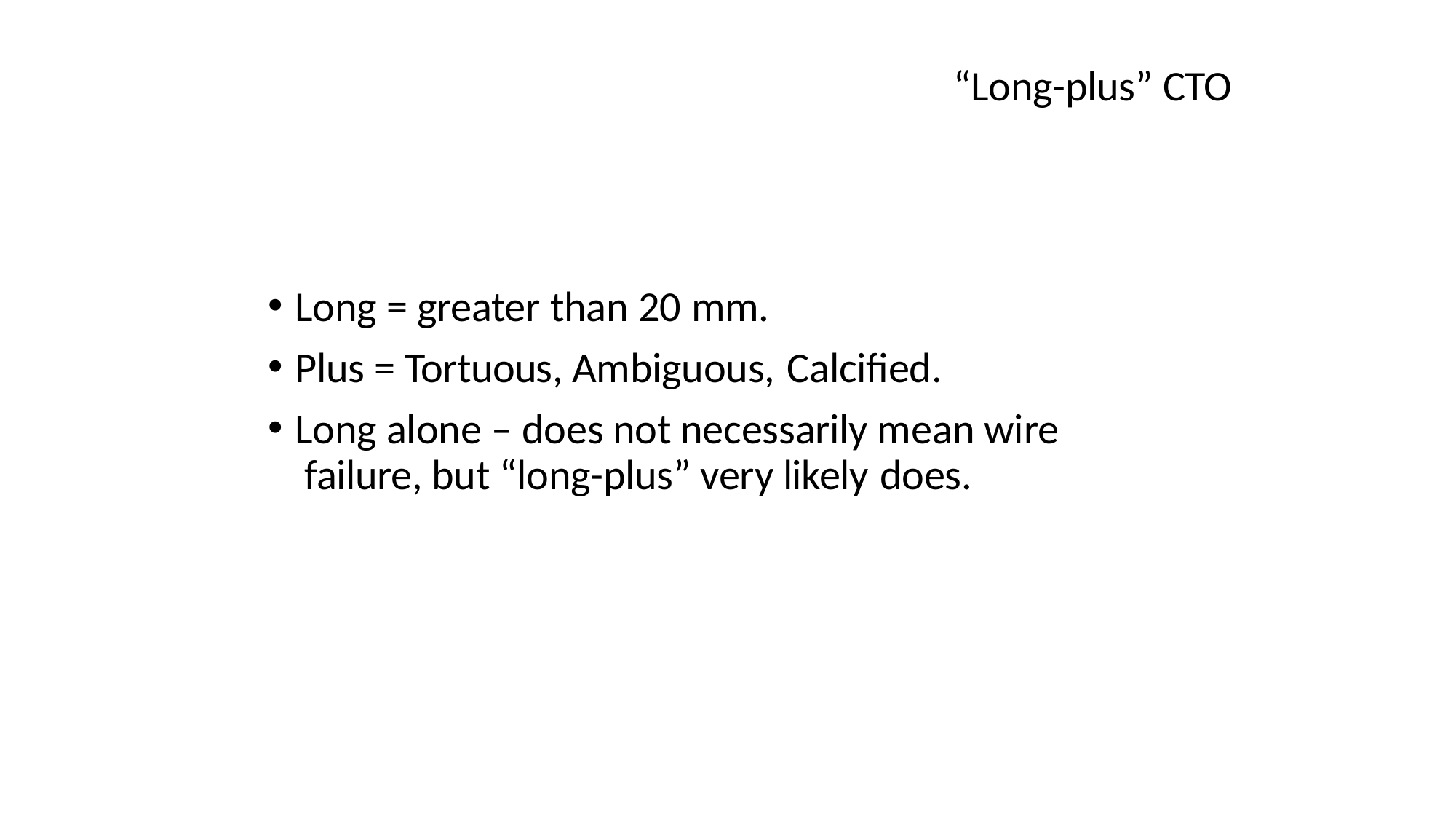

# “Long-plus” CTO
Long = greater than 20 mm.
Plus = Tortuous, Ambiguous, Calcified.
Long alone – does not necessarily mean wire failure, but “long-plus” very likely does.

## Slide 21
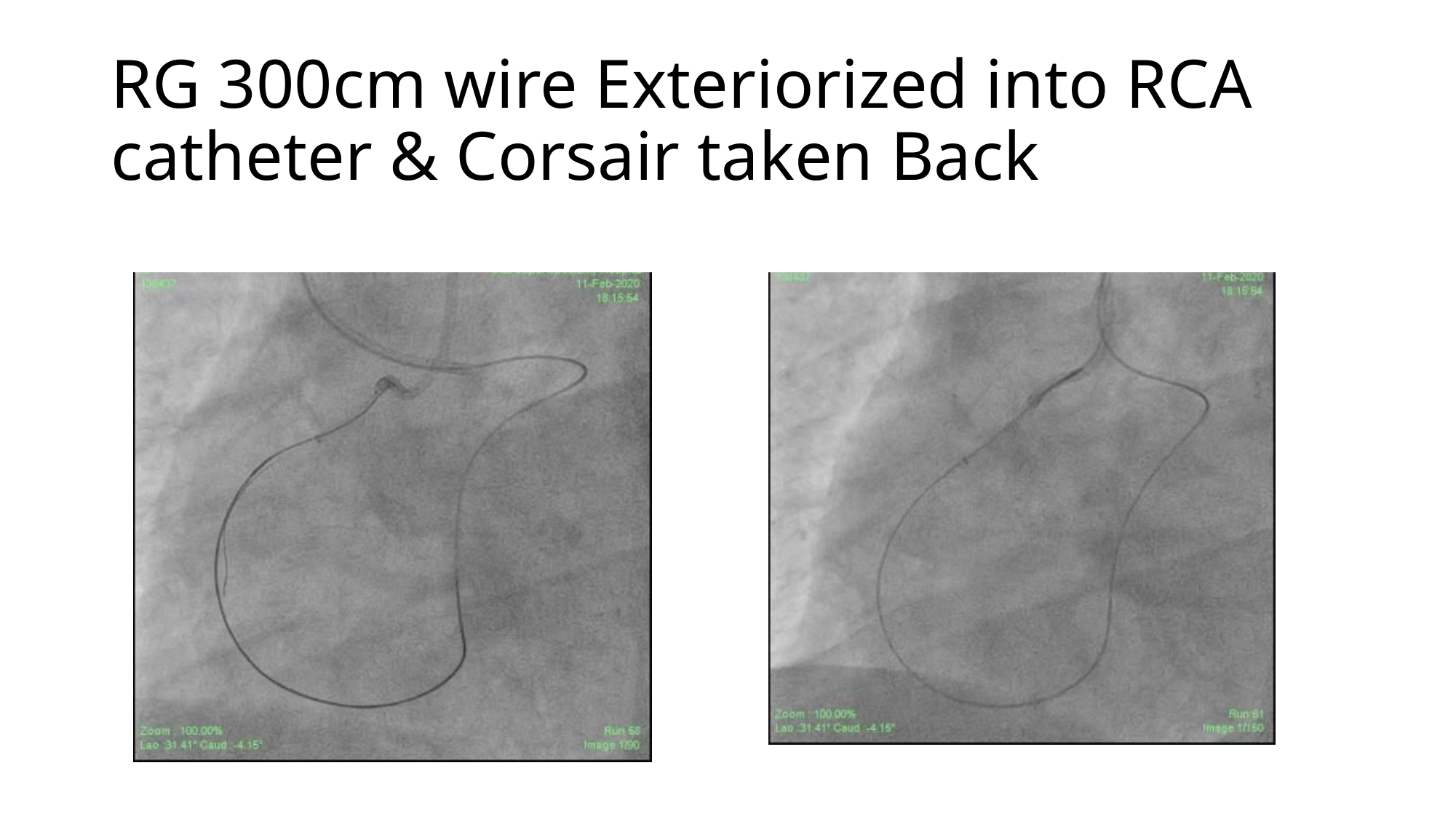

# RG 300cm wire Exteriorized into RCA catheter & Corsair taken Back

## Slide 22
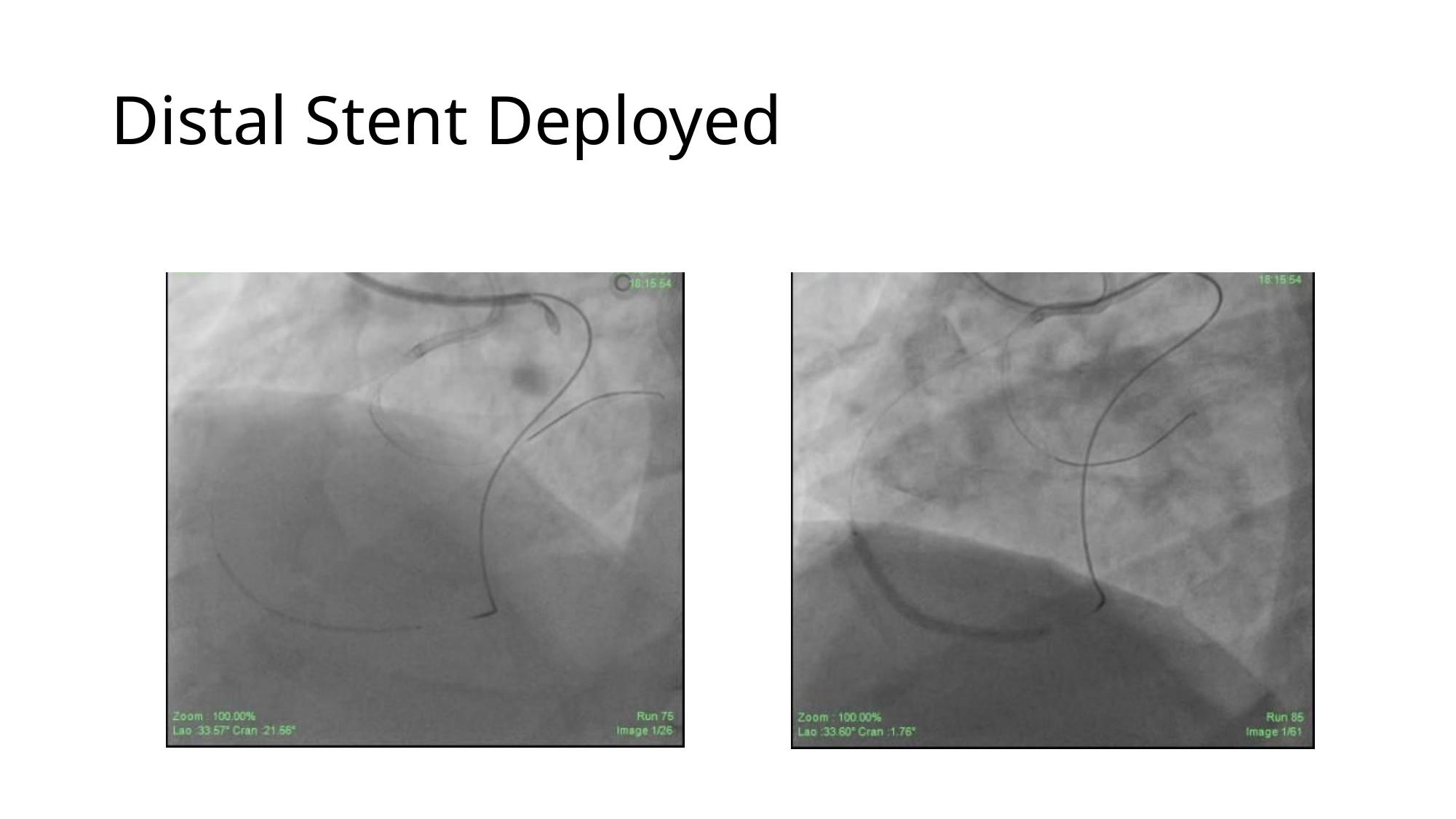

# Distal Stent Deployed

## Slide 23
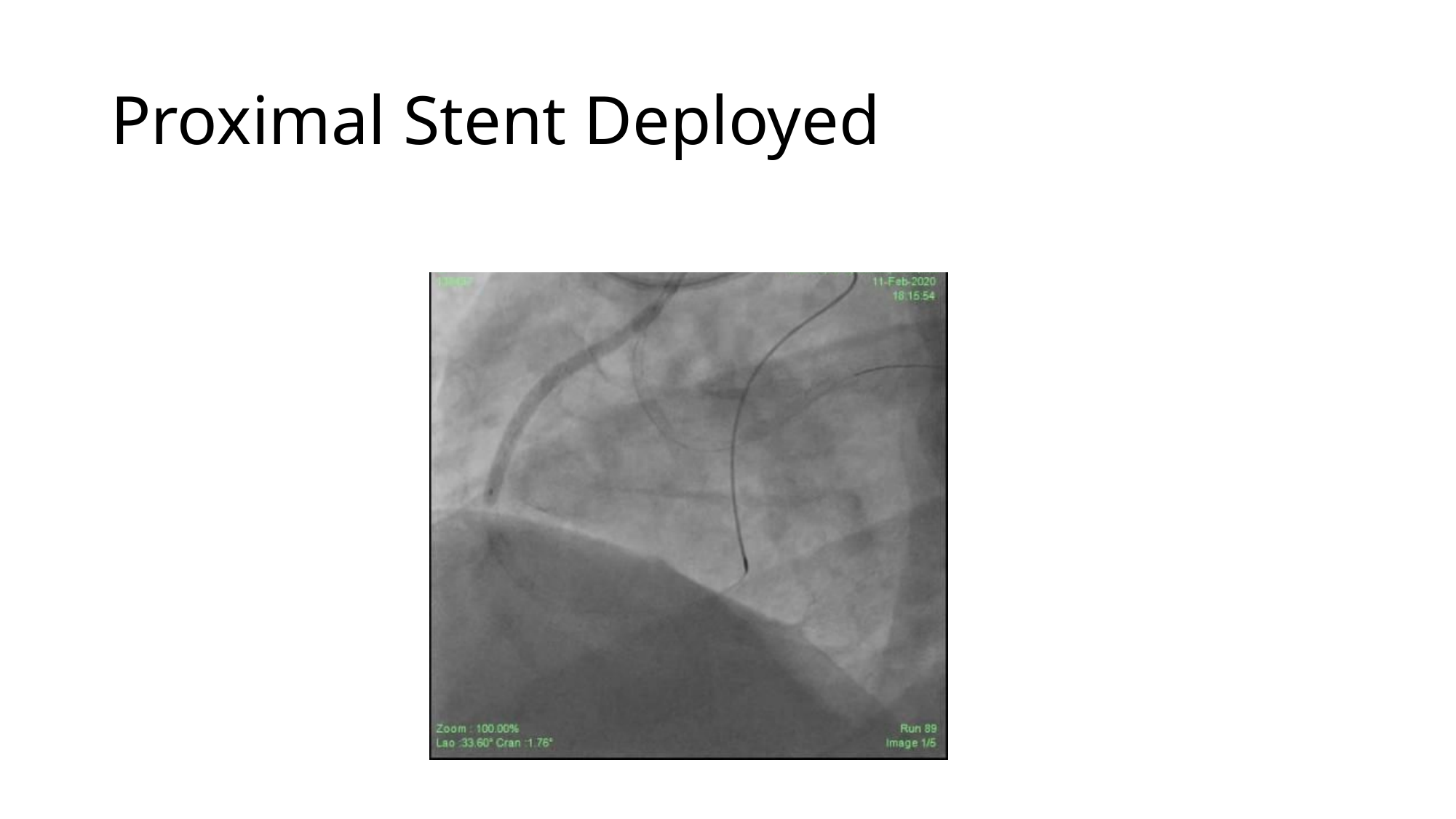

# Proximal Stent Deployed

## Slide 24
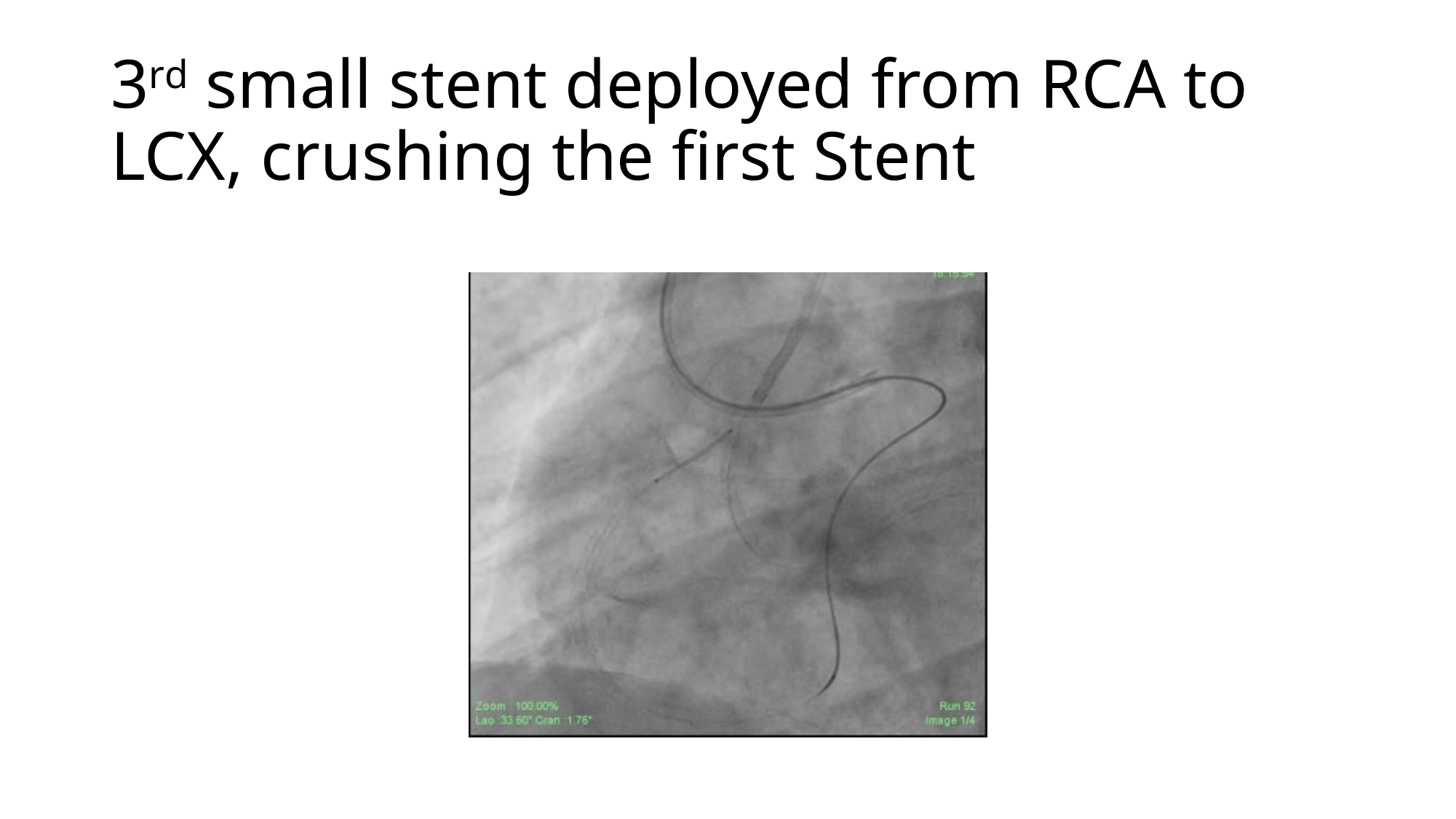

# 3rd small stent deployed from RCA to LCX, crushing the first Stent

## Slide 25
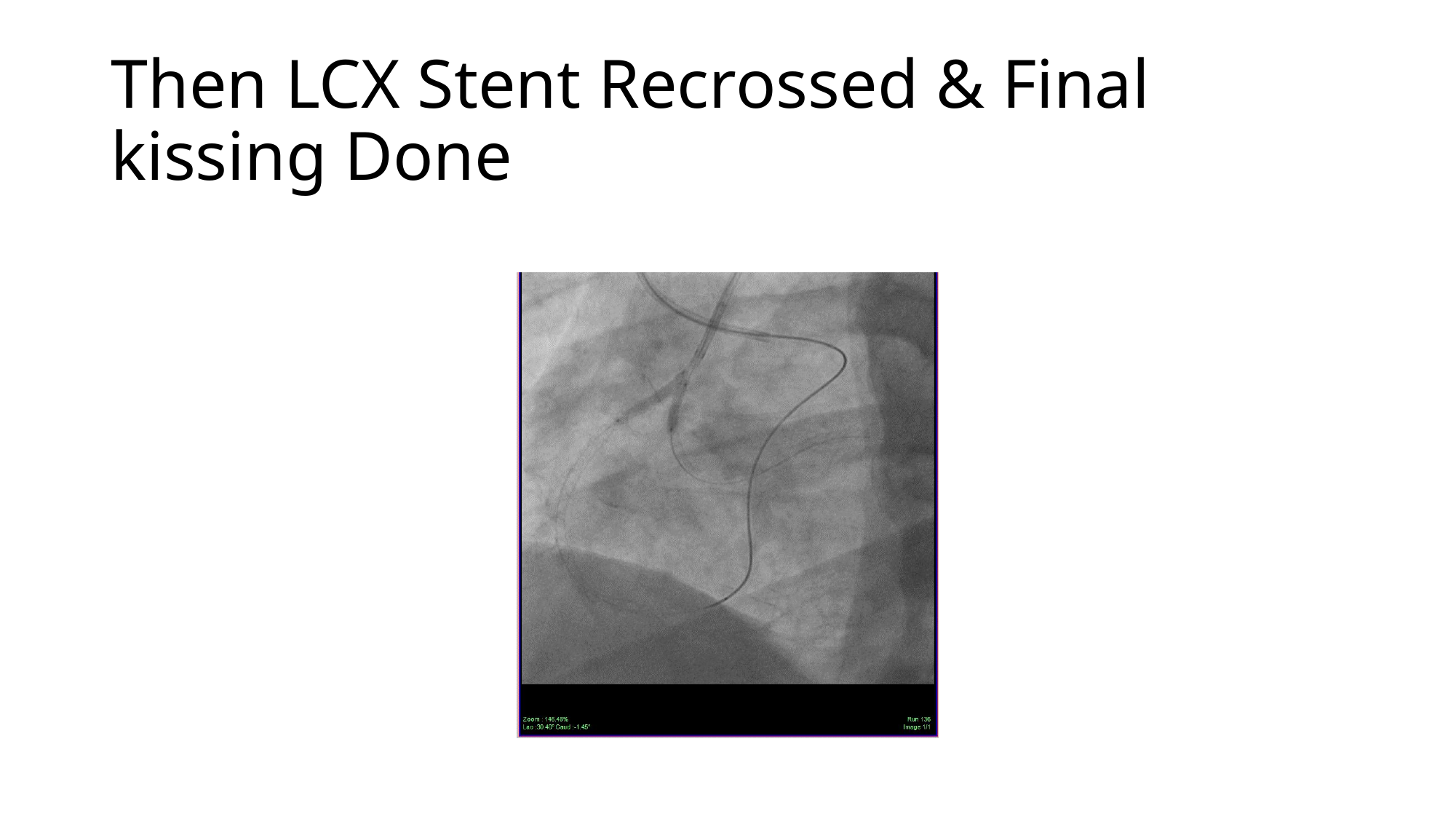

# Then LCX Stent Recrossed & Final kissing Done

## Slide 26
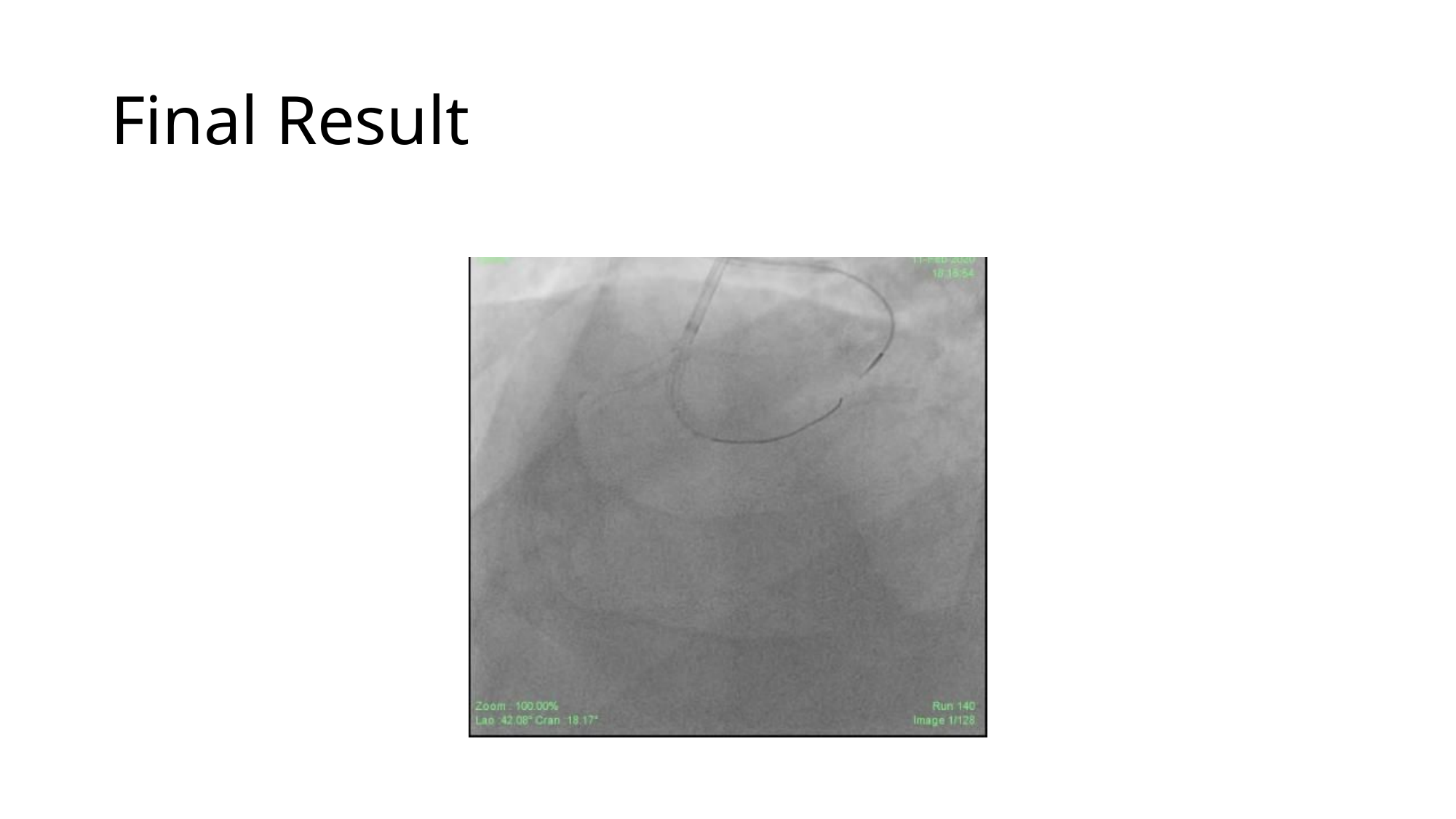

# Final Result

## Slide 27
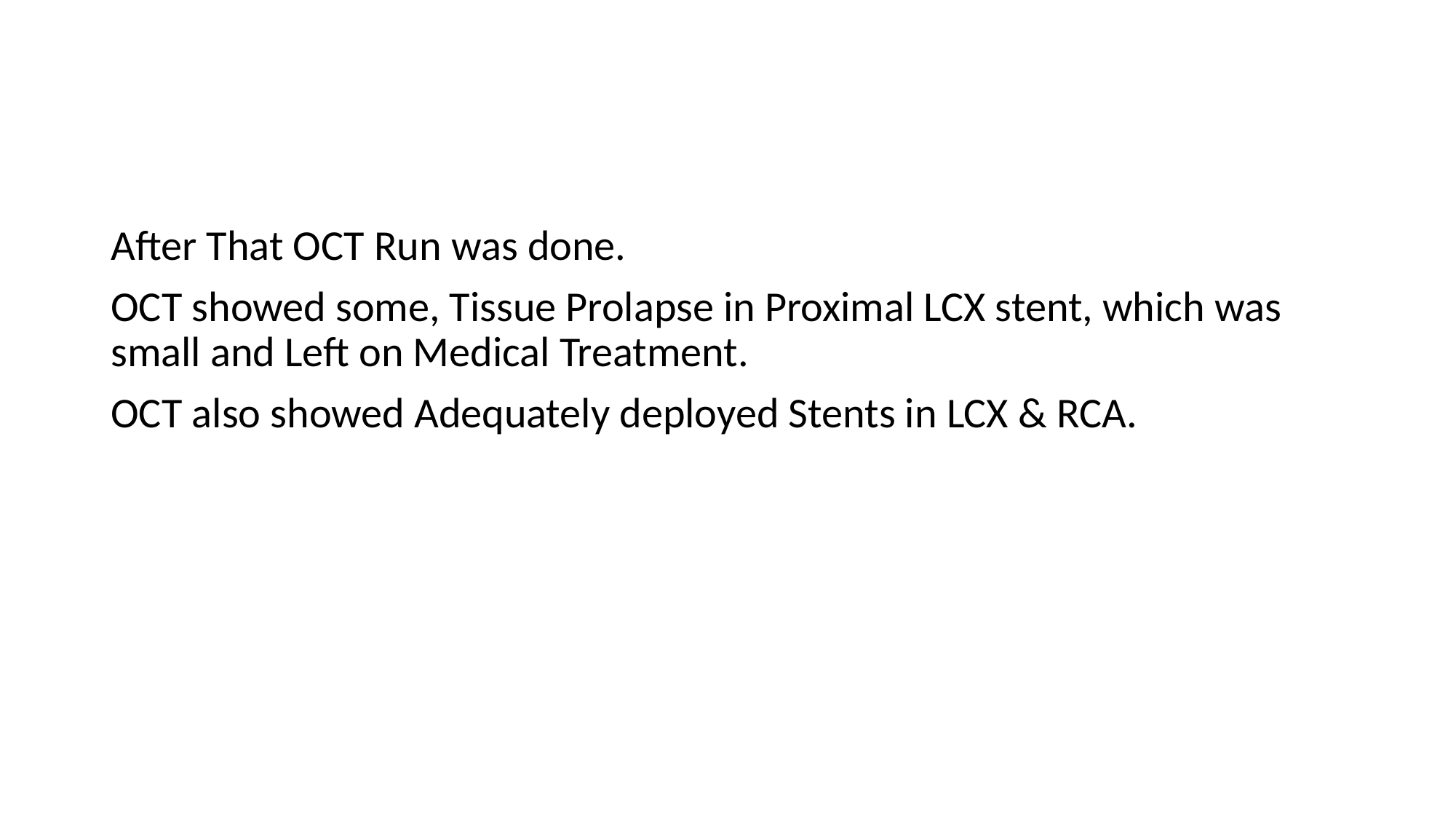

#
After That OCT Run was done.
OCT showed some, Tissue Prolapse in Proximal LCX stent, which was small and Left on Medical Treatment.
OCT also showed Adequately deployed Stents in LCX & RCA.

## Slide 28
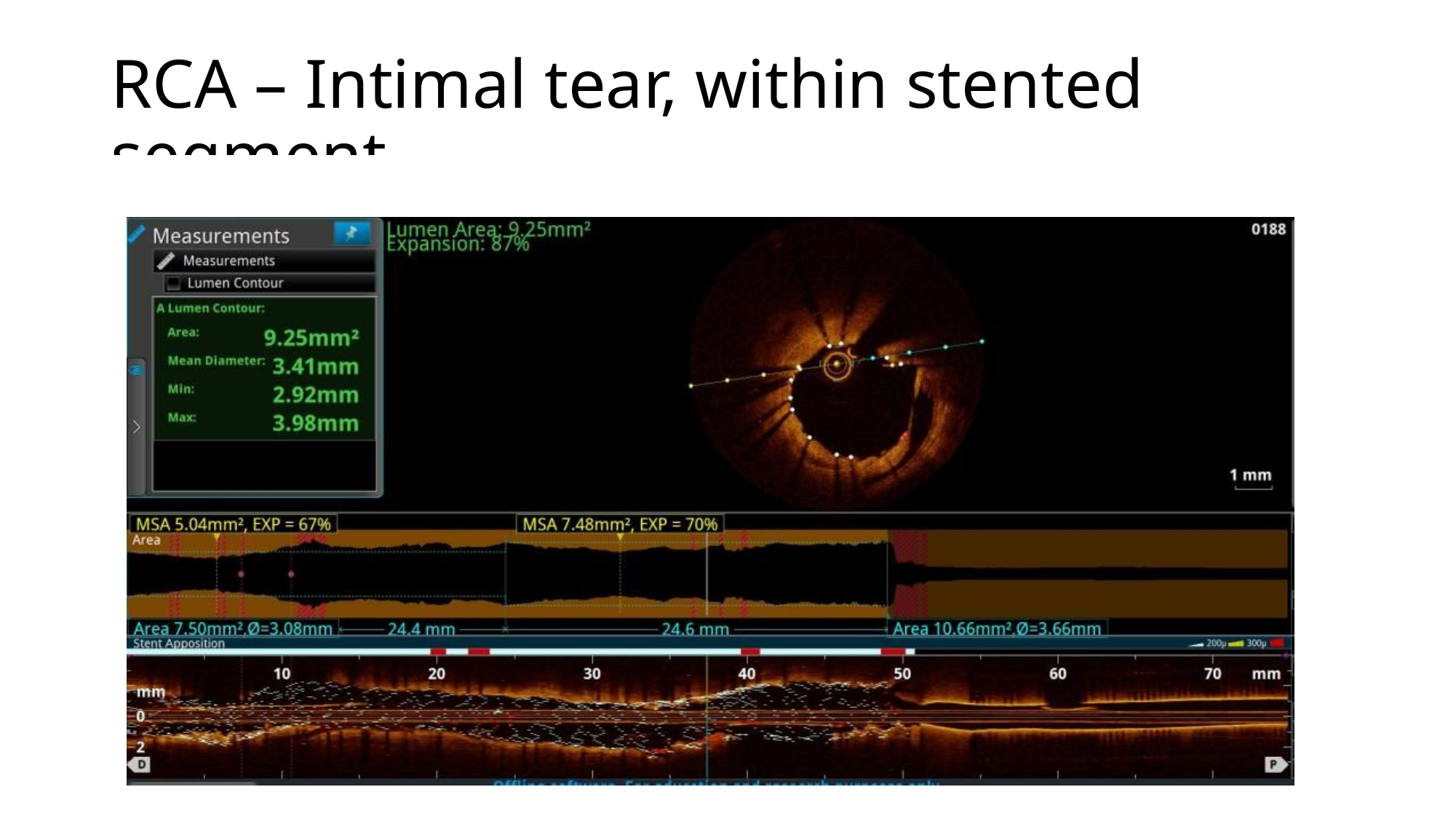

# RCA – Intimal tear, within stented segment.

## Slide 29
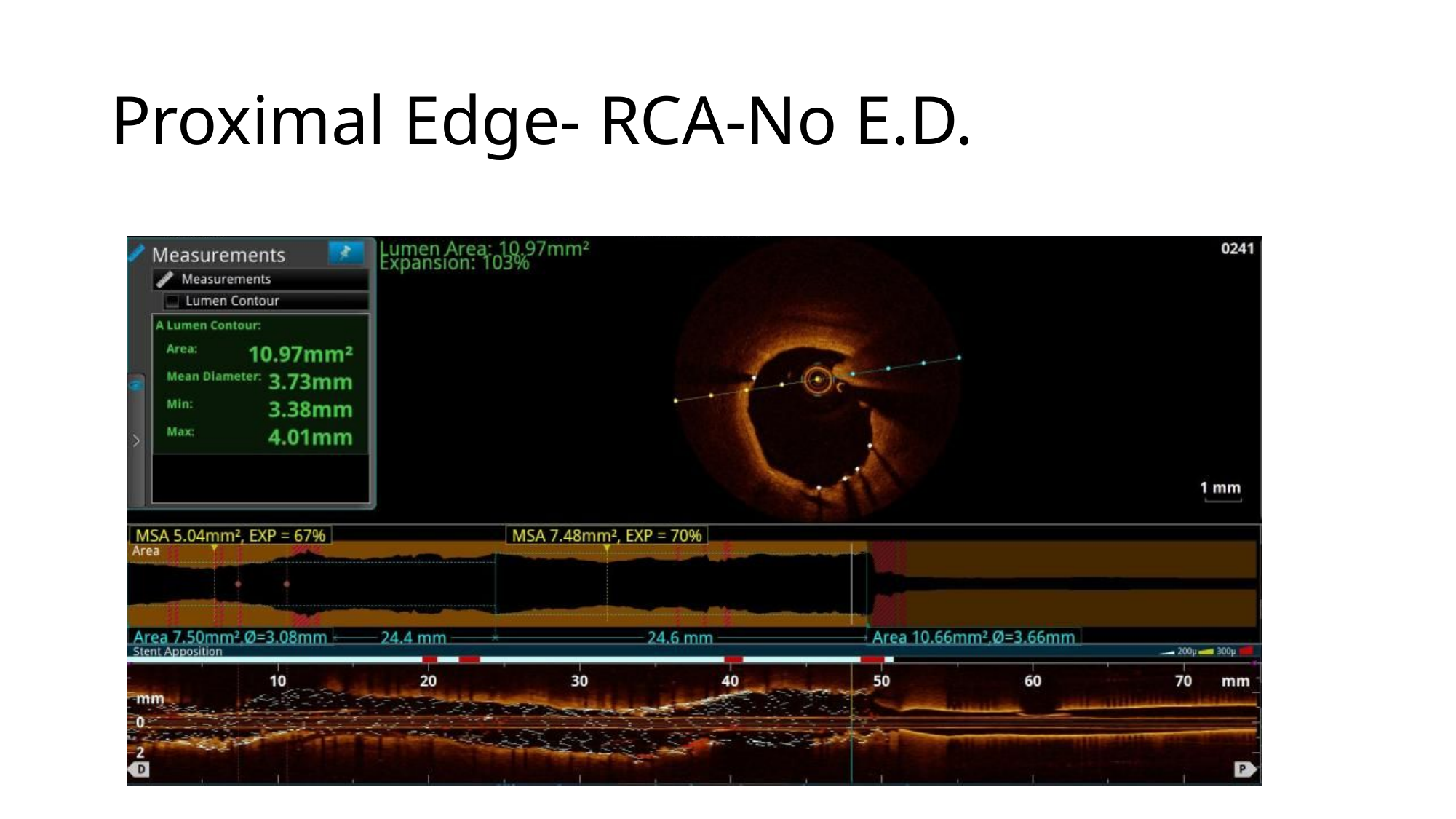

# Proximal Edge- RCA-No E.D.

## Slide 30
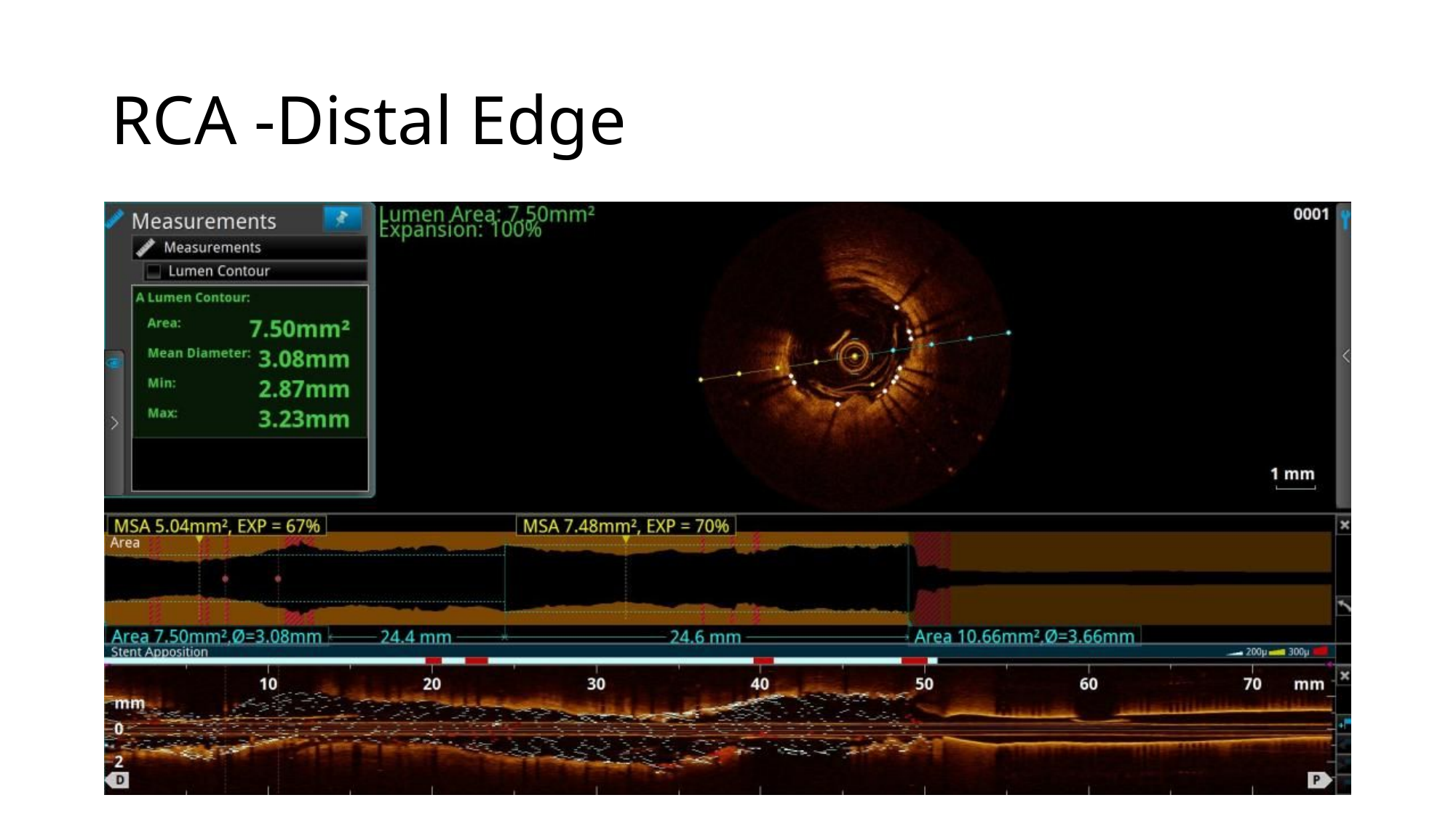

# RCA -Distal Edge

## Slide 31
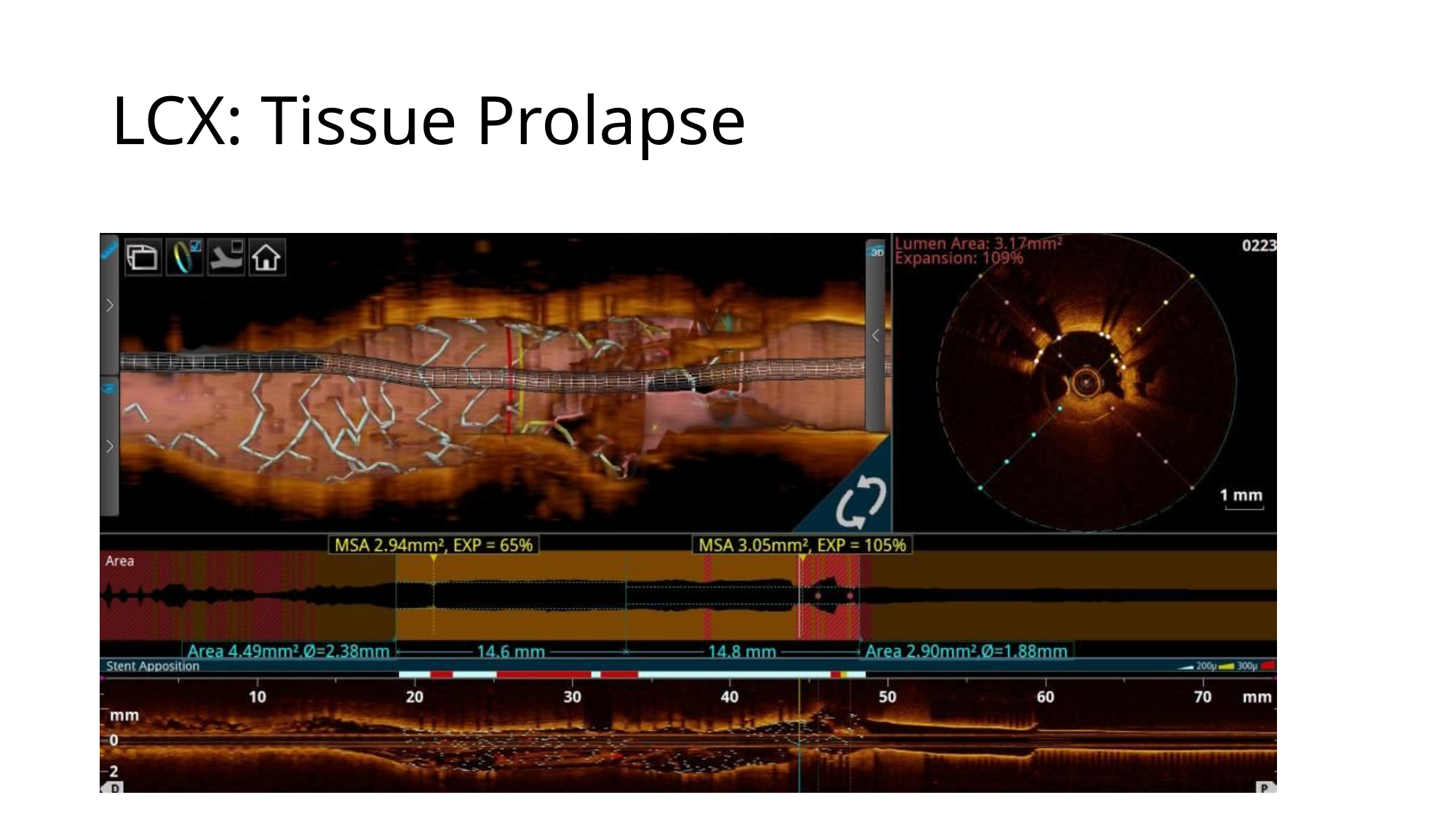

# LCX: Tissue Prolapse

## Slide 32
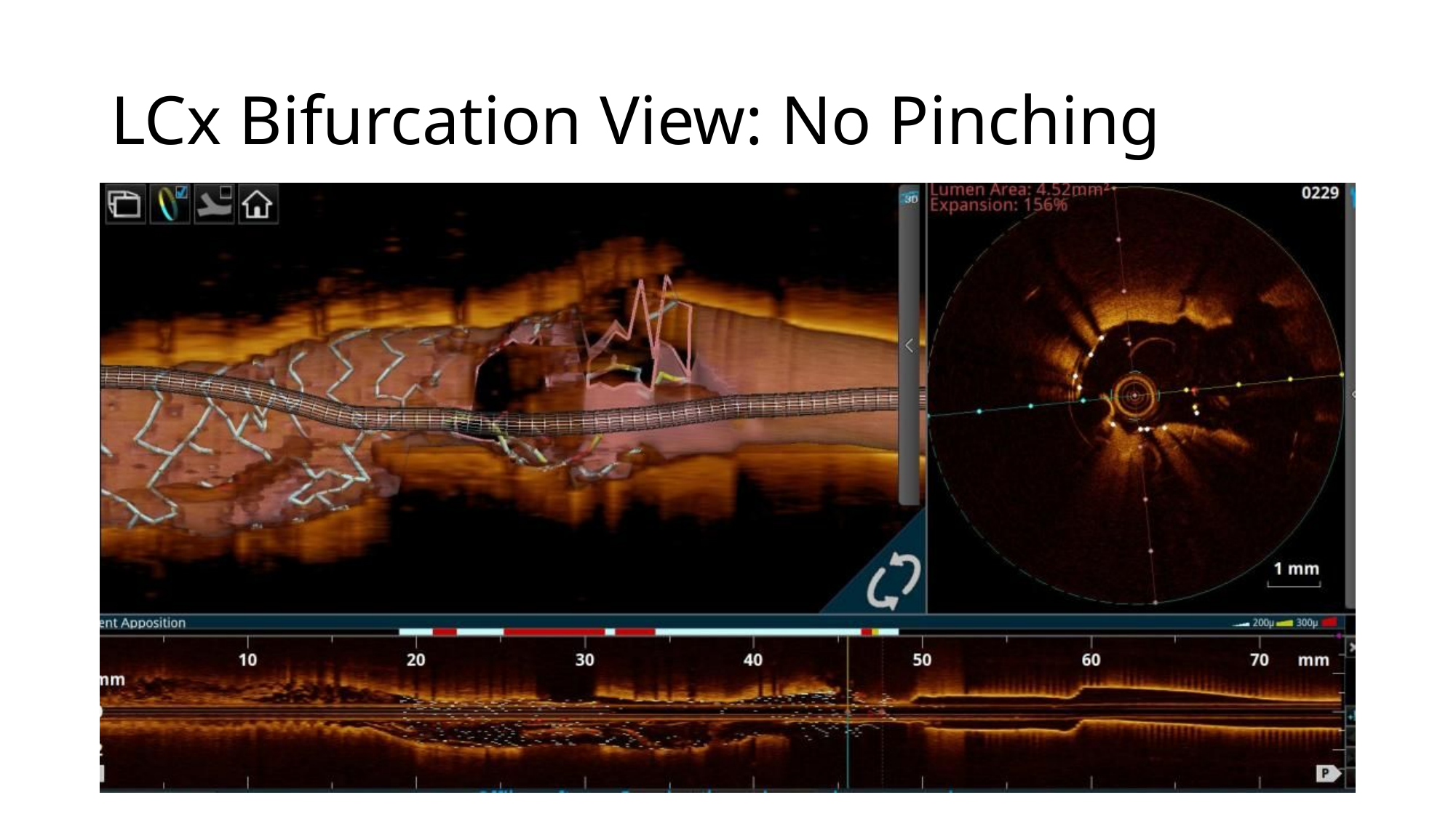

# LCx Bifurcation View: No Pinching

## Slide 33
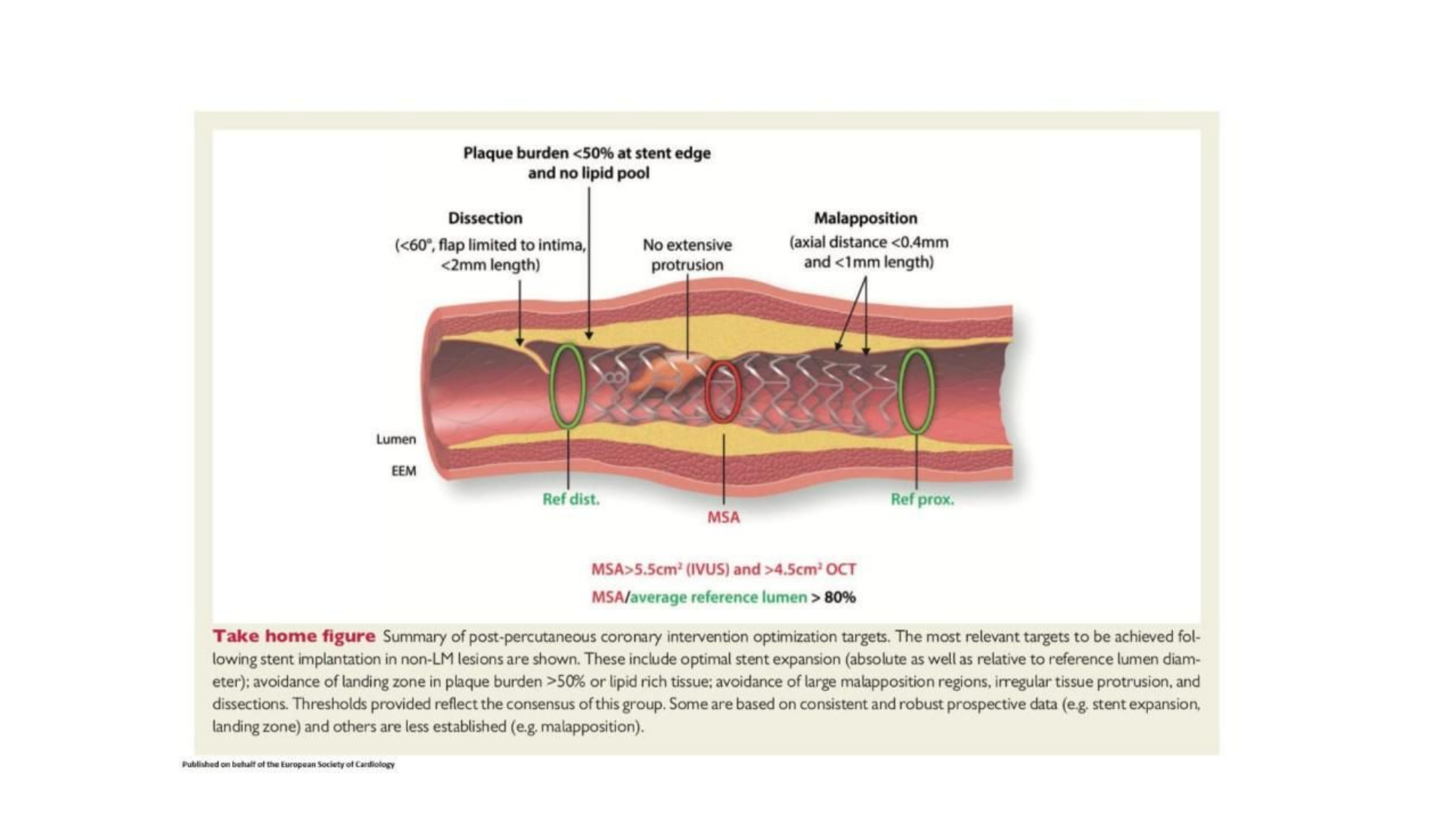

#
